# Societies of strangers do not speak less complex languages

**DOI:** 10.1126/sciadv.adf7704

**Published:** 2023-08-16

**Authors:** Olena Shcherbakova, Susanne Maria Michaelis, Hannah J. Haynie, Sam Passmore, Volker Gast, Russell D. Gray, Simon J. Greenhill, Damián E. Blasi, Hedvig Skirgård

**Affiliations:** ^1^ Department of Linguistic and Cultural Evolution, Max Planck Institute for Evolutionary Anthropology, Leipzig 04103, Germany.; ^2^ Department of Linguistics, University of Colorado Boulder, Boulder, CO 80309, USA.; ^3^ Evolution of Cultural Diversity Initiative, School of Culture, History and Language, College of Asia and the Pacific, The Australian National University, Canberra, ACT 2601, Australia.; ^4^ Department of English and American Studies, Friedrich-Schiller University of Jena, Jena 07745, Germany.; ^5^ School of Psychology, University of Auckland, 1010 Auckland, New Zealand.; ^6^ School of Biological Sciences, University of Auckland, 1010 Auckland, New Zealand.; ^7^ Department of Human Evolutionary Biology, Peabody Museum, Harvard University, Cambridge, MA 02138, USA.; ^8^ Human Relations Area Files, Yale University, New Haven, CT 06520, USA.

## Abstract

Many recent proposals claim that languages adapt to their environments. The linguistic niche hypothesis claims that languages with numerous native speakers and substantial proportions of nonnative speakers (societies of strangers) tend to lose grammatical distinctions. In contrast, languages in small, isolated communities should maintain or expand their grammatical markers. Here, we test these claims using a global dataset of grammatical structures, Grambank. We model the impact of the number of native speakers, the proportion of nonnative speakers, the number of linguistic neighbors, and the status of a language on grammatical complexity while controlling for spatial and phylogenetic autocorrelation. We deconstruct “grammatical complexity” into two separate dimensions: how much morphology a language has (“fusion”) and the amount of information obligatorily encoded in the grammar (“informativity”). We find several instances of weak or moderate positive associations but no inverse correlations between grammatical complexity and sociodemographic factors. Our findings cast doubt on the widespread claim that grammatical complexity is shaped by the sociolinguistic environment.

## Teaser

Spatiophylogenetic modelling allows testing the claim that grammatical complexity is reduced in societies of strangers.

## INTRODUCTION

Societies vary greatly in their size, homogeneity, and degree of contact with other societies. The variation in these properties is captured in the continuum between two extremes: “societies of intimates” (esoteric societies) and “societies of strangers” (exoteric societies) (*1*–*4*). Societies of intimates are small-sized tight-knit homogenous groups where members share high amounts of knowledge about community life and do not engage much with outsiders (*1*, *2*, *5*–*8*). On another extreme, we encounter societies of strangers: large heterogeneous groups with substantial proportions of outsiders (either people using a different language or at least outsiders to the local community), loose networks, and, as a result, lower amounts of shared communal information. We refer to this continuum that spans from societies of intimates to societies of strangers as exotericity. Within this continuum, societies low in exotericity are prototypical societies of intimates (esoteric societies), and those high in exotericity correspond to the characteristics of societies of strangers.

Different degrees of exotericity in societies have been hypothesized to shape the communication between individuals—ultimately resulting in observable effects on grammatical structures. Two prominent pathways link exotericity and language structure. First, the members of homogenous esoteric societies rarely communicate with outsiders, and hence, the languages in such societies are acquired and used almost exclusively by members of these societies. This lack of contact with nonnative speakers has been claimed to shape languages such that they develop and retain more obligatory explicit grammatical markings (*1*, *5*, *9*). For example, Tariana, an endangered Arawakan language in the Amazonas has an evidential marking system: The verbs carry grammatical information that distinguishes between situations where the speaker has seen or heard the action they report, or where this action is inferred or assumed from second- or third-hand information (*10*). This grammatical feature has been suggested to occur in low-exotericity languages rather than highly exoteric ones (*11*).

Second, the social setting of exoteric communities with high proportions of outsiders and degrees of contact with nonnative L2 (second language) speakers has been proposed to drive morphological simplification in languages (*5*, *6*). L2 speakers find it especially difficult to process and produce phonologically fused grammatical structures, such as case endings and verbal agreement markers (*5*, *6*, *12*, *11*, *13*). Hence, these languages have been suggested to undergo a process of simplification, such as the loss of morphological categories and agreement. For instance, since the Old English period, English has lost the adjective agreement in case, number, and gender as well as the nominal case distinctions, which has been linked to the adoption of English by nonnative speakers (*11*). Similarly, it has been proposed that gender systems, another feature more typical of low-exotericity languages (*11*), tend to reduce in languages that undergo contact with other languages, especially those without gender, i.e., when the societies speaking these languages become more exoteric (*14*), or even disappear, as in Ossetic and Cappadocian Greek, where the loss of gender has also been linked to L2 learning and contact (*15*). Apart from adult L2 speakers failing to faithfully learn a foreign language, the simplification (loss or reduction of phonologically fused marking) can result from L1 (first language) speakers consciously or unconsciously accommodating their speech to the needs of the outsiders by reducing grammatical markers that pose acquisition difficulties (*5*).

These links between language and social structure have received a substantial amount of attention, mainly through the lens of focused, small-scale comparisons. A range of qualitative studies has analyzed closely related varieties of Quechua (*8*), English (*11*, *16*), and German (*17*, *18*) as well as Tibeto-Burman (*19*) and Scandinavian languages (*7*). These studies seem to corroborate that, among closely related varieties, the languages that were more exposed to contact with L2 speakers tend to show less irregular, less opaque grammatical markers in the studied domains.

However, the extent to which these findings generalize beyond a handful of cases remains unclear. Each of these studies calls on different (and sometimes idiosyncratic) linguistic and sociodemographic variables, which calls into question the homogeneity of the causes and the mechanisms underlying them. This limited comparability was partially addressed by comparative studies that aimed at assessing these hypotheses at a global scale. Lupyan and Dale (*6*) showed that different aspects of morphological complexity are inversely correlated with population size (the number of L1 speakers), geographic spread, and the number of linguistic neighbors, which became known as the linguistic niche hypothesis. A follow-up study (*20*) found no correlation between the number of cases in nouns and population size but showed that this linguistic feature is negatively correlated with the proportion of L2 speakers. Yet, other studies (*21*) reveal a negative correlation between verbal synthesis and both demographic variables (the number of L1 speakers and the proportion of L2 speakers) within the same model. Last, some studies find no relationship between morphological complexity and the presence or absence of a substantial proportion of L2 speakers (*22*). All in all, it remains unclear whether any of these measures of exotericity are meaningfully associated with language structure. Large language samples in these works made it challenging to reliably place the studied communities on an exotericity continuum. Reliable information on all criteria (homogeneity of the population, community size, social network density, relative isolation, etc.) delineating the distinctions between more and less exoteric communities was unavailable, so instead different sociodemographic variables served as proxies for exotericity.

The inconsistent findings of previous studies may have arisen for three reasons. First, the cross-linguistic coverage of these studies varies substantially and, thus, raises the question of how representative these samples are of global differences in grammatical complexity. The limited sample size often results from uneven feature coverage in the WALS (The World Atlas of Language Structures) database (*23*). WALS covers 2662 languages but only a few hundred have information available for more than 50% of features. This makes studying multiple features associated with complexity impossible without decreasing the sample or having more uncertainty in the data. For instance, the problem of data sparseness affects the morphological complexity scores presented in (*6*), which are calculated for all languages with at least 3 out of 28 features. At the same time, the language samples used for most analyses of individual grammatical features in the same study are also modest: The median value is 218 languages per feature. Second, the previous studies involve widely different linguistic phenomena that are assumed to be comparable only through the lens of the umbrella term “grammatical complexity.” Grammatical complexity has many facets: the number of markers, irregularity, obligatoriness, compositionality, redundancy, and reliance on phonologically fused rather than independent forms (*5*, *6*, *24*, *25*). The multifaceted nature of complexity means that a language is seen as more complex as it increases the number of grammatical cases and determiners, irregular verb forms, noncompositional constructions, agreement patterns, and/or phonologically fused markers expressing different functions. However, different underlying mechanisms can be responsible for the changes in these distinct dimensions of complexity in exoteric societies. For example, when complexity is viewed in terms of compositionality, language structures are claimed to become more compositional (they consist of several interpretable parts rather than one independently interpretable part) in exoteric societies where high proportions of L2 speakers benefit from additional transparency (*5*). These different dimensions can all change at different rates and under different pressures. Hence, combining several of these dimensions into one metric of grammatical complexity [e.g. (*22*, *26*)] may not shed light on the relationship between grammatical structures and the chosen sociodemographic variables reflecting exotericity.

To test the association between grammatical structures and exotericity of the relevant societies, we introduce metrics that quantify two distinct dimensions of grammatical complexity that have been claimed to be reduced in exoteric societies: (i) the degree of phonologically fused grammatical markers (“fusion”) and (ii) the number of obligatory grammatical marking (“informativity”). We obtain grammatical features included in both metrics from the large global dataset Grambank (*27*, *28*) (see table S1 for the list of Grambank features in both metrics).

The fusion score reflects the degree to which the languages rely on phonologically bound markers (e.g., prefixes and suffixes) as opposed to phonologically independent markers. While phonologically independent markers are independent of other morphemes in their stress and form, phonologically fused markers rely on other morphemes in this respect, which makes the task of acquiring phonologically fused markers by adult L2 learners more difficult. Languages with phonologically fused markers for tense-aspect-mood categories on verbs, case on nouns and pronouns, gender and number agreement, possession, negation, and other features will score higher on this metric. For instance, the highest-scoring language, Tariana (Arawakan), has overt morphological plural marking on nouns; core and oblique cases on nouns and pronouns; overt morphological marking of mood, aspect distinctions, present, past, and future tenses; morphological passive on verbs; and number and gender agreement on different targets, among others.

The informativity metric offers insight into the amount of obligatory explicit grammatical distinctions made by languages. The following features, for example, increase informativity: politeness distinction in pronouns, remoteness distinctions in past and future tenses, definite and indefinite articles, and number marking on nouns (singular, dual, plural, trial, paucal; associative plural). Languages will score higher on the informativity metric if their grammars have, for instance, different demonstratives used for visible and nonvisible objects, as in Tundra Nenets (Uralic), where *tay°kuy° teda* “that reindeer” would refer to the visible reindeer and *t′exa teda* “that reindeer” would be used when the reindeer is not visible, for example, when it is behind something (*29*). We have not included features in this metric if they concerned distinctions that are generally considered to be universal, such as negation (*30*) and possession (*31*). We are unaware of any language that does not make a distinction between affirmative and negated clauses nor any that lack a productive pattern at all for marking possession. Including these features would not tell us anything meaningful about variation in the expression of grammatical meaning in the world’s languages.

Another feature that contributes to informativity is the presence of distinctions between inclusive and exclusive constructions in pronominal systems or verbal indexing. For instance, Māori (Austronesian) disambiguates two potential meanings of “we” left unspecified in English: To produce a sentence *We will go on a walk*, the speakers of Māori obligatorily choose between *tātou* if the interlocutor is joining and *mātou* if the interlocutor is not included in “we” and the speaker goes on a walk with other people (*32*). Apart from excluding the features from the metric that are marked in all or almost all languages, since Grambank was designed to capture features found commonly in the world’s languages, our metric does not contain any extremely rare features marked by only a handful of languages. The informativity metric is not sensitive to whether the information is marked by a fused marker or not.

The third potential reason for inconsistent findings in the previous studies is that they do not fully control for phylogenetic and spatial nonindependence [but see (*33*)], although both factors can influence the interpretation of the results. For instance, the predictive power of most negative relationships between WALS and population size has been shown to reduce after shuffling the languages within families (*6*). Specifically, the previous studies control for these confounds in a way that implies assembling languages into large groups based on their ancestry and locations, which oversimplifies the relationships between them. Except for (*33*) and (*34*), previous studies tend to treat membership in the same language family as a random effect. However, this approach ignores the relationships between the languages within the same families. Alternatively, the previous studies sample languages from different families and locations. However, this sampling does not always ensure the independence of data points (*35*, *36*) and invariably leads to more constrained samples with a subsequent loss in statistical power (*37*). Similarly, random effects of geographical areas such as Glottolog’s macroareas or the 24 AUTOTYP areas (*38*) are used to control for spatial nonindependence. With large macroareas, all languages spoken in different continents are grouped together and the differing effect of distance between two neighboring languages and two languages on different sides of the continent are neglected. Random effects of detailed areas have the same problem that individual distances between languages within the same area do not inform the analysis. In addition, this does not capture the contact between neighboring languages if they belong to two different areas. For example, although Ukrainian and Polish are closely related geographical neighbors, they are modeled as belonging to distinct areas (Inner Asia and Europe) when AUTOTYP areas are modeled as random effects.

### Testing the hypothesis

Here, we test the hypothesis that languages in highly exoteric societies have (i) fewer phonologically fused grammatical markers (fusion) and (ii) overall fewer obligatory explicit markers (informativity) compared to languages in low-exotericity societies. We aim to overcome past limitations by (i) using a comprehensive and diverse sample of the world’s languages exceeding those in previous studies, (ii) motivating the variables involved in the relation between exotericity and language structure, and (iii) crafting a state-of-the-art statistical model accounting for the complex historical dependencies between languages.

#### Languages and societies sample

**Fig. 1.**
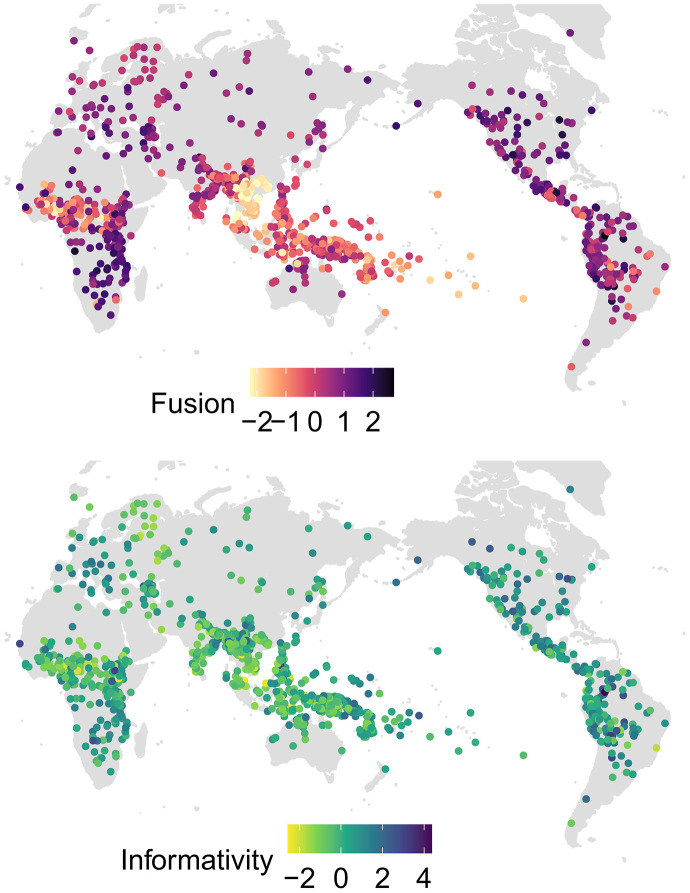
The global distribution of fusion and informativity scores. The scores with a minimum of 0 (absence of all metric features) and a maximum of 1 (presence of all metric features) have been standardized to a mean of 0 and a variance of 1. The hotspots of low fusion are located in West Africa and Southeast Asia. Many Austronesian languages also rank low on fusion. The geographic patterns of informativity scores are less clear compared to fusion. Among lower-scoring languages are those spoken in West Africa, Southeast Asia, many Uralic languages, and languages spoken in India (Indo-Aryan and Dravidian).

**Fig. 2.**
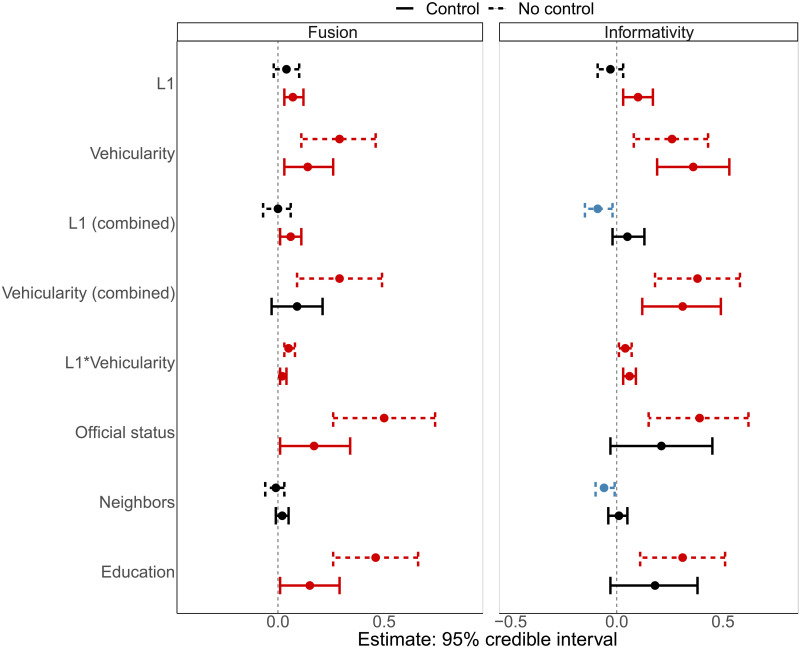
The coefficients and 95% credible intervals for fixed effects in six bivariate regression models and one multivariable model (L1 combined and Vehicularity combined) for fusion and informativity, without and with spatiophylogenetic random effects (dashed and solid lines respectively). The linear regression coefficients of fixed effects representing exotericity in the spatiophylogentic models are depicted with the error bars. Error bars in black cross zero, whereas the bars colored red and blue indicate robust positive and negative relationships, respectively. Many effects that appear influential (colored in red or blue) in models that do not control for random effects of genealogy and geography (dashed error bars) disappear after we control for these sources of nonindependence (solid error bars). The exceptions are the weak positive effects of L1 speakers on fusion and informativity that are revealed in the full model with fixed and random effects but remain hidden in the model where the number of L1 speakers is the only predictor of metric scores. Further exceptions are the weak or moderate positive effects of Vehicularity on fusion and informativity and official status and education use on fusion. This shows that controlling for the nonindependence of languages is indispensable for unraveling the proposed relationships of dependence between grammatical structures and sociodemographic factors. The short names of the effects of the number of L1 speakers and Vehicularity differ based on the model that they are incorporated in: The models where these variables are modeled in isolation [L1 (log-transformed and standardized number of L1 speakers) and Vehicularity effects], the combined model with both of these effects [L1 (combined) (log-transformed and standardized number of L1 speakers) and Vehicularity (combined)], and the model with an interaction term (L1*Vehicularity), where the log-transformed number of L1 speakers was used.

Our sample consists of 1291 languages (see Figs. 1 and 2). The majority of these languages belong to the following language families: Austronesian (291), Sino-Tibetan (144), Atlantic-Congo (140), Afro-Asiatic (57), Austroasiatic (53), and Indo-European (44). Most languages in our sample are located in Oceania (212), Southeast Asia (155), African Savannah (136), and the Indian subcontinent (106). Indo-European languages are not overrepresented in our large-scale sample, which is a persistent problem in many cross-linguistic studies.

#### Sociodemographic variables of exotericity

We examine whether the variation in the scores of fusion and informativity is explained by the sociodemographic factors associated with more exotericity of the societies. This dimension is complex and so far it has escaped simple quantification; however, there are a number of globally available social and demographic variables that correspond to different degrees of exotericity. In general, society is considered to be more exoteric, if the following metrics are larger or if the binary variables are present:

 1) Number of L1 speakers [cf. (*6*, *21*)]
 2) Vehicularity (as an indicator of the proportion of L2 speakers) [cf. (*20*–*22*)]
 3) Number of linguistic neighbors [cf. (*6*)]
 4) Official status [cf. (*39*)]
 5) Usage in education

The last two variables concerning the status of the language (official/not official; used or not used in education) have rarely been used in predicting grammatical structures. We include these because they help to capture another side of exotericity: Exoteric communities are more likely to use languages that are recognized as official languages and languages of education. We predict that these two variables, official status and usage in education, play a crucial role in the linguistic niche hypothesis. They either enable the written form to become more elaborate while the spoken form simplifies (*40*) or they should mitigate the hypothesized negative effect of the number of L1 speakers on grammatical complexity. Both of these factors should act to make the dominant language more conservative, thus preventing the loss of complex features and increasing their transmission fidelity. In addition, recent studies (*41*) have indicated that language of education, in particular, is a major cause of minority language loss which will strengthen the selective pressure to learn the dominant language.

Vehicularity has been argued to be a reliable indicator of whether a language is expected to have L2 speakers (*22*). This variable was constructed following the approach described in (*22*) based on the Expanded Graded Intergenerational Disruption Scale (EGIDS) available in the Ethnologue (*57*). The EGIDS scale reflects how endangered a language is; level 0 stands for “International”, and level 10 stands for “Extinct”. If a language has a high EGIDS level, such as 0 (“International”), 1 (“National”), 2 (“Provincial”) or 3 (“Wider Communication”), the language is considered “vehicular”, i.e. expected to have L2 speakers. In contrast, languages of level 4 (“Educational”) and higher (“Developing”, “Vigorous”, “Threatened”, “Shifting”, “Moribund”, “Nearly Extinct”, “Dormant”, and “Extinct”) are not likely to be used by L2 speakers.

All variables are modeled in isolation from each other because of the high probability of multicollinearity: A language with many L1 speakers is more likely to have more linguistic neighbors and act as an official language and a language of education.

Although it has been suggested that larger populations are more likely to have higher proportions of L2 speakers (*6*), it is possible that societies with similar population sizes might still differ in proportions of L2 speakers, which will have different implications for the evolution of these languages if the link between exotericity and grammatical structures holds true. For instance, a society with a large L1 speaker population and few L2 speakers will be lower on the exotericity scale than a language with a similar population size but an extreme proportion of L2 speakers. Because of this and the importance of accounting for multiple linguistic and social factors (*21*, *42*), we additionally fit two models that use the number of L1 speakers and Vehicularity as (i) two separate fixed effects and (ii) as an interaction term between them. On a subsample of 120 languages with available data from Ethnologue for calculating proportion of L2 speakers, we fit the same set of models with proportion of L2 speakers instead of Vehicularity.

#### Spatiophylogenetic modeling

Using a Bayesian phylogenetic framework, we map fusion and informativity scores obtained from Grambank with available information about the locations from Glottolog 4.5 (*43*) and the sociodemographic variables to the global tree (*44*) of EDGE (evolutionarily distinct, globally endangered) languages. As a result, we have a global sample of 1291 languages available on the phylogeny for which both metric scores were calculated and sociodemographic data was present.

We adopt spatiophylogenetic modeling (*45*) that allows us to study the relation between sociodemographic and linguistic factors while taking into account the complex spatial and genealogical relations between languages and societies. Both spatial and genealogical relations are represented as random effects built on the basis of covariance matrices that stand for the relevant historical processes.

First, we fit different combinations of random effects to determine whether the distribution of fusion and informativity scores depends on the phylogenetic and geographical dependencies of the languages. In this step, we build seven models containing the intercept and random effects as predictors:

Phylogenetic effects

 1) Spatial effects: “local” diffusion of scores across several hundreds of kilometers is possible
 2) Spatial effects: “regional” diffusion of scores across distances up to 1000 km (see Methods and Materials and the “Spatial effects” section and fig. S1 for more details)
 3) Spatial effects: 24 language areas from AUTOTYP
 4) Phylogenetic effects + spatial effects: local
 5) Phylogenetic effects + spatial effects: regional
 6) Spatial effects: 24 language areas from AUTOTYP

Second, we choose the strongest model to test whether adding sociodemographic variables to it will improve its fit. This implies fitting seven models with different sociodemographic variables or their combinations treated as fixed effects:

 1) Number of L1 speakers
 2) Vehicularity
 3) Number of L1 speakers and Vehicularity (combined model) [cf. (*21*)]
 4) The interaction term between these two variables (number of L1 speakers * Vehicularity)
 5) Number of linguistic neighbors
 6) Official status (binary)
 7) Language of education (binary)

We fit these seven models without any random effects to compare to which extent controlling for nonindependence influences the results. Then, we compare the models of fusion and informativity to determine the influential predictors of the metric scores. We compare all competing models in our analyses based on obtained widely applicable information criterion values [WAIC; (*46*, *47*)].

## RESULTS

Out of the set of random effects models, both fusion and informativity scores are best predicted by the combination of phylogenetic and spatial effects (Tables 1 and 2). The spatiophylogenetic models incorporating both random effects substantially outperform other models, in particular, the runner-up phylogenetic-only models. This indicates that both effects explain variation in the scores better than phylogenetic effects in isolation. The differences between WAIC values (*46*) between the strongest spatiophylogenetic models and other models are larger than 45 for both fusion and informativity. The preference for the local as opposed to the regional version of the spatial random effect suggests the likely diffusion of the scores across short distances of several hundreds of kilometers. Whereby the random effects assuming a much wider possible diffusion, i.e., regional spatial effects (>1000 km) or the random effects of 24 language areas, performed worse.

**Table 1.** WAIC values and quantiles (0.025, 0.5, and 0.975) of estimates of models fitting only random effects and intercept in models predicting fusion. Bolded text indicates effects are substantial (do not include zero).

Model	Effect	2.5%	50%	97.5%	WAIC
Phylogenetic + spatial: local + L1 speakers	**Phylogenetic SD**	1.53	1.75	2.02	1796.34
	**Spatial SD**	0.28	0.34	0.41	
	Intercept	−0.02	0.00	0.02	
	**L1**	0.03	0.07	0.12	
Phylogenetic + spatial: local + L1 speakers + Vehicularity	**Phylogenetic SD**	1.53	1.75	2.02	1798.71
	**Spatial SD**	0.27	0.34	0.40	
	Intercept	−0.04	−0.01	0.02	
	**L1**	0.01	0.06	0.11	
	Vehicularity	−0.03	0.09	0.21	
Phylogenetic + spatial: local + L1_log10:Vehicularity	**Phylogenetic SD**	1.52	1.74	2.01	1814.65
	**Spatial SD**	0.27	0.34	0.40	
	Intercept	−0.04	−0.01	0.01	
	L1*Vehicularity	0.01	0.02	0.04	
Phylogenetic + spatial: local + Vehicularity	**Phylogenetic SD**	1.52	1.74	2.01	1814.82
	**Spatial SD**	0.27	0.34	0.40	
	Intercept	−0.04	−0.01	0.01	
	**Vehicularity**	0.03	0.14	0.26	
Phylogenetic + spatial: local + Neighbors	**Phylogenetic SD**	1.52	1.74	2.01	1818.78
	**Spatial SD**	0.27	0.34	0.41	
	Intercept	−0.03	0.00	0.02	
	Neighbors	−0.01	0.02	0.05	
Phylogenetic + spatial: local + Official	**Phylogenetic SD**	1.50	1.72	1.99	1820.45
	**Spatial SD**	0.28	0.34	0.41	
	Intercept	−0.03	−0.01	0.02	
	**Official status**	0.01	0.17	0.34	
Phylogenetic + spatial: local + Education	**Phylogenetic SD**	1.50	1.72	1.99	1821.36
	**Spatial SD**	0.28	0.34	0.41	
	Intercept	−0.04	−0.01	0.01	
	**Education**	−0.01	0.15	0.29	

**Table 2.** WAIC values and quantiles (0.025, 0.5, and 0.975) of estimates of models fitting only random effects and intercept in models predicting informativity. Bolded text indicates effects are substantial (do not include zero).

Model	Effect	2.5%	50%	97.5%	WAIC
Phylogenetic + spatial: local + L1 speakers + Vehicularity	**Phylogenetic SD**	0.92	1.24	1.60	3128.91
	**Spatial SD**	0.36	0.44	0.53	
	Intercept	−0.04	0.00	0.04	
	L1	−0.02	0.05	0.13	
	**Vehicularity**	0.12	0.31	0.49	
Phylogenetic + spatial: local + L1 speakers	**Phylogenetic SD**	0.97	1.28	1.66	3132.76
	**Spatial SD**	0.35	0.43	0.53	
	Intercept	−0.01	0.03	0.07	
	**L1**	0.03	0.10	0.17	
Phylogenetic + spatial: local + L1_log10:Vehicularity	**Phylogenetic SD**	0.89	1.20	1.57	3135.58
	**Spatial SD**	0.36	0.44	0.54	
	Intercept	−0.05	0.00	0.04	
	L1*Vehicularity	0.03	0.06	0.09	
Phylogenetic + spatial: local + Vehicularity	**Phylogenetic SD**	0.87	1.18	1.55	3136.39
	**Spatial SD**	0.36	0.44	0.54	
	Intercept	−0.05	0.00	0.04	
	**Vehicularity**	−0.19	0.36	0.53	
Phylogenetic + spatial: local + Neighbors	**Phylogenetic SD**	0.88	1.20	1.57	3153.06
	**Spatial SD**	0.36	0.44	0.54	
	Intercept	−0.01	0.03	0.07	
	Neighbors	−0.04	0.01	0.05	
Phylogenetic + spatial: local + education	**Phylogenetic SD**	0.85	1.15	1.54	3156.96
	**Spatial SD**	0.37	0.45	0.54	
	Intercept	−0.02	0.02	0.06	
	Education	−0.03	0.18	0.38	
Phylogenetic + spatial: local + official	**Phylogenetic SD**	0.83	1.14	1.52	3157.39
	**Spatial SD**	0.37	0.45	0.54	
	Intercept	−0.02	0.02	0.06	
	Official status	−0.03	0.21	0.45	

This best-fitting model incorporating phylogenetic and spatial effects is then used to test whether adding any of the sociodemographic predictors (or their combinations) contributed to understanding the distribution of fusion and informativity. We find that the effects of these predictors range from negligible to low. The strongest models predicting fusion and informativity are those incorporating these random effects, the number of L1 speakers, and Vehicularity. The models including other social variables are comparable with the spatiophylogenetic models.

However, the linear regression coefficients of many fixed effects are negligible: They either overlap with or are not appreciably different from zero (see Fig. 2). This also applies to the results on the subsample of 120 languages with available data on proportion of L2 speakers which reveal no evidence that this variable is correlated with fusion or informativity (see table S2). Only some tested sociodemographic variables are weak or moderate predictors of metrics scores. For fusion, we find a weak positive correlation with the number of L1 speakers variable in two models where it is (i) the only sociodemographic variable and (ii) in combination with Vehicularity (the 95% credible intervals for the effects of L1 speakers on fusion in both models: 0.03 to 0.12 and 0.01 to 0.11, respectively). Similarly, official status and use in education show a weak positive correlation with fusion when used as the only predictors in the models (the 95% credible intervals: 0.01 to 0.34 and 0.01 to 0.29, respectively). For informativity, we find a weak positive effect of the number of L1 speakers (the 95% credible intervals: 0.03 to 0.17) only when it is the only sociodemographic variable in the model. This effect disappears (the 95% credible intervals: −0.02 to 0.13) when both the number of L1 speakers and Vehicularity are included in the same model (the 95% credible intervals for the effects of Vehicularity in this combined model on informativity: 0.12 to 0.49). In addition, we find a weak positive effect of the interaction between the number of L1 speakers and Vehicularity on both fusion and informativity (the 95% credible intervals: 0.01 to 0.04 and 0.03 to 0.09, respectively). None of these relationships are negative as predicted by prior studies. This contradicts the argument advanced by the linguistic niche hypothesis that there should be an inverse relationship between grammatical complexity and the sociodemographic factors associated with exotericity.

All models with fixed effects that exclude random effects of phylogenetic and spatial relationships rank lower than the same models that additionally implement random effects (see table S2). This means that the predictive performance of models without random effects is inferior compared to the models that incorporate both fixed and random effects.

Instead, the distribution in fusion and informativity scores is largely explained by phylogenetic random effects—92 and 68% of the total variance of fusion and informativity—and spatial random effects that account for 4 and 9% of the total variance of the scores correspondingly (see table S3). We measure the phylogenetic signal of both complexity dimensions on the global tree by estimating Pagel’s lambda (λ) (*48*). The values of this metric range from 0, which indicates no phylogenetic signal (random distribution of scores with respect to the phylogeny), to 1, which indicates a strong phylogenetic signal (closely related languages share similar scores), or greater than 1. Both fusion and informativity show strong phylogenetic signals: The phylogenetic signal of fusion (λ = 0.97, <0.001) is stronger than that of informativity (λ = 0.86, <0.001). In other words, fusion and informativity scores are explained by the inheritance from a common ancestor and spatial diffusion among close neighbors. Nevertheless, the role of spatial relationships might have been downplayed as the information about the locations of languages has informed the structure of the global EDGE tree, in that weak geographic priors were imposed on likely language relationships within the phylogeographic model. This means that the relative contribution of the spatial predictor might be larger.

One alternative explanation for why we find no substantial effect of exotericity on fusion and informativity is that these relationships are nonlinear. It might be the case, for instance, if the effects of the number of L1 speakers on grammatical structures are only substantial for extremely large or extremely small communities. To address this possibility, apart from fitting linear regressions with the number of L1 speakers, we operationalize this variable in the form of nonlinear effects within the random walk models of order 2 (RW2). We find that these models rank similarly to their counterparts with corresponding linear effects. Since the SD of these random nonlinear effects is small (<0.04 for fusion and informativity), we report only the results of linear regression models in the main text and provide table S2 summarizing the WAIC values and effects of all fitted models in the Supplementary Materials.

## DISCUSSION

The specific claim of the “linguistic niche hypothesis” that grammatical complexity should reduce with an increased number of nonnative speakers is not supported by our results. Contrary to the expected inverse correlation between complexity scores and sociodemographic variables reflecting exotericity, the only effects we find are weakly or moderately positive.

**Fig. 3.**
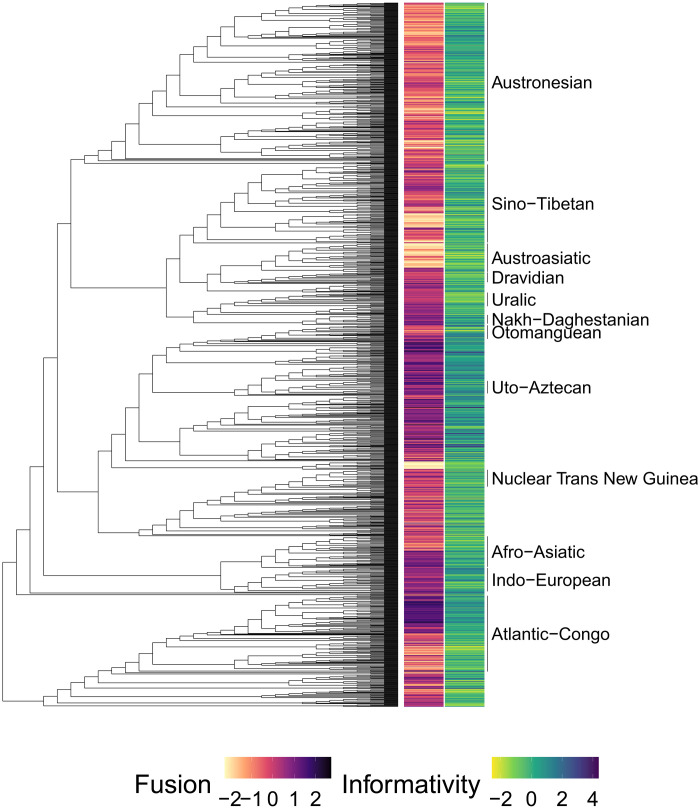
The scores of fusion and informativity on the global tree. The scores with a minimum of 0 (absence of all metric features) and a maximum of 1 (presence of all metric features) have been standardized to a mean of 0 and a variance of 1. We detect many patterns of closely related languages scoring similarly, which might indicate the faithful transmission of grammatical complexity from ancestor languages to their descendants rather than large-scale adaptations of grammatical complexity to changes in sociodemographic factors. Similar to geographic distribution, we see that fusion scores follow a more defined pattern of phylogenetic clustering compared to informativity scores.

Instead, we found that the two dimensions of complexity we model—fusion and informativity—are better predicted by genealogy and geographic diffusion than by exotericity measures. Measuring the phylogenetic signal of these two features also showed that the distribution of their scores was largely influenced by the shared evolutionary histories between languages on the global tree. Both of these dimensions of grammatical complexity appear to be highly stable phylogenetically (see Fig. 3), which suggests that fusion and informativity are phylogenetically constrained.

**Fig. 4.**
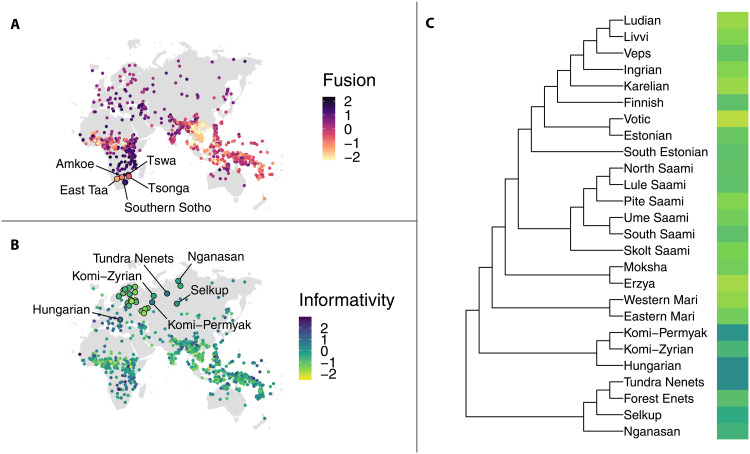
The global distribution of fusion and informativity scores and the distribution of fusion scores on the subset of the global tree. The panels display the distribution of fusion scores in Southern Africa (**A**) and informativity scores in Eurasia (**B**) with a focus on Uralic languages and the phylogeny of Uralic languages included in the global tree (**C**). The scores with a minimum of 0 (absence of all metric features) and a maximum of 1 (presence of all metric features) have been standardized to a mean of 0 and a variance of 1. The difference in fusion scores between two Bantu languages—Tsonga (low) and Southern Sotho (high)—can be explained from the perspective of phylogenetic relatedness, with Tsonga resembling the scores of other low-scoring outliers in Southern Africa from other language families than Bantu and to lesser extent its neighboring sister language Tswa (A). Higher informativity scores in Uralic languages show a clear pattern of phylogenetic clustering (C) and are found in Samoyedic (Nganasan, Selkup, Tundra Nenets, and Forest Enets), Ugric (Hungarian), and Permian (Komi-Permyak and Komi-Zyrian), which diverged earlier from the rest of the languages. Higher informativity scores in Uralic languages can also be ascribed to contact with Indo-European languages (B): Hungarian is surrounded by higher-scoring languages spoken in Europe and the speakers of Tundra Nenets and Komi-Permyak are typically bilingual in Russian.

Taking a closer look at the languages spoken in Southern Africa shows why phylogenetic distances explain the variation in fusion scores better than the demographic properties of the societies (see Fig. 4A). Most languages in this area have high fusion scores, including the high-scoring language, Southern Sotho (1.38): a Southern Bantu language used in a highly exoteric society by >5.5 million L1 speakers and >7 million L2 speakers. By contrast, Tsonga, another Southern Bantu language, scores notably low: −0.6. It is also spoken in an exoteric niche, but its degree of exotericity is lower since it has fewer L2 speakers (>3 million) than Southern Sotho, although it has approximately 1 million more L1 speakers. Given their sociolinguistic environments, we could expect more resemblance in the scores between these two languages and more fusion in Tsonga. However, we observe a less pronounced difference between Tsonga’s score and that of its neighboring sister language Tswa with an above-average (0.7) score, spoken in a more esoteric niche by >6 million L1 speakers and no L2 speakers. Some other languages with notably low fusion scores in Southern Africa are languages from other language families: East Taa (Tuu) (−1.43) with 2500 L1 speakers and severely endangered Amkoe (Kxa) (−0.94) with several dozen L1 speakers. Despite being spoken in highly esoteric societies, these languages rank extremely low on fusion. The fact that out of the examined languages in Southern Africa, the lower scoring ones (or moderately scoring ones as is the case for Tswa) are either two closely related Bantu languages or languages from language families other than Bantu indicates that genealogical relationships and to some extent potential contact between neighboring languages provide a better basis for understanding the variation in fusion scores.

The Uralic family exemplifies a case where phylogenetic effects might be challenging to disentangle from spatial ones because the distribution of informativity scores can be explained by both phylogenetic relationships and geographic distances/contact phenomena. Most Uralic languages score low on the informativity metric (see Fig. 4, B and C). The exceptions with higher scores are languages belonging to branches such as Samoyedic (Nganasan, Selkup, Tundra Nenets, and Forest Enets), Ugric (Hungarian), and Permian (Komi-Permyak and Komi-Zyrian), which diverged earlier from the rest of the languages subsumed under the Mari, Mordvin, Saami, and Finnic branches (*46*). The contrasts in informativity scores between these sister languages do not seem to match the hypothesis that lower informativity should exist in exoteric societies. For instance, Votic with 25 speakers scores lower in informativity (−1.79) than its closest relative Estonian (−0.8) with >1 million speakers. Although phylogenetic distances serve as an explanation for the distribution of the scores in the family, geographic proximity/contact may have been another influential factor. For instance, Hungarian matches the scores of the surrounding higher-scoring Indo-European languages. Similarly, Tundra Nenets and Komi-Permyak might score high in informativity due to the bilingualism of their speakers in Russian. Unlike higher proportions of adult L2 speakers, child bilingualism has been associated with an increase in grammatical complexity rather than its loss (*11*).

One of the key differences between the analysis presented here and previous studies is that we use spatiophylogenetic methods to explicitly model the effects of genealogical and geographic nonindependence. This allows us not only to address two sources of nonindependence between languages in our cross-linguistic samples (*41*, *49*) but also to quantify and compare the relative importance of the phylogenetic and spatial effects. The majority of previous studies ignore the dependence between languages belonging to the same family or located in the same area by treating families and areas as random effects, or sampling languages from distinct families and locations in an attempt to exclude languages that are nonindependent (*35*, *36*). Our results clearly show that this methodological difference really matters (Fig. 2). Only some variables, like the number of L1 speakers and Vehicularity along with official status and use in education for fusion, emerge weak or moderate positive effects after controlling for genealogy and geography, whereas other effects appear influential only in models without these random effects and disappear when the nonindependence of languages is controlled for.

To examine the differences between our results and findings reported in the previous studies, we reanalyzed the morphological complexity data used in (*6*) [obtained from G. Lupyan, personal communication 02 June 2023, and made available in the web-archive Zenodo (https://doi.org/10.5281/zenodo.10420654) in the data folder as complexity_data_WALS.csv]. We modeled the relationship between the morphological complexity scores and the number of L1 speakers. The results revealed a negative but extremely weak correlation between morphological complexity and the number of L1 speakers in the model with spatiophylogenetic effects (−0.02). No correlation was found in the model without these effects. To see whether these findings of reanalysis are sensitive to the cutoff criterion for the minimum feature coverage [the morphological complexity scores in (*6*) are calculated if at least 10% of metric features are available in WALS for a language], we raise the cutoff to the minimum of 35% of available features. We find no effect of the number of L1 speakers on morphological complexity with the raised cutoff while controlling for spatiophylogenetic effects (see table S4). Given these findings, it is expected that, when modeling our fusion score (calculated using the data from another database) with the number of L1 speakers as the only predictor (not using any control for genealogy and geography), we find no evidence for a correlation between these variables. This suggests that the fundamental difference between our results and the findings of the previous studies likely lies both in the data used for calculating grammatical complexity scores and the proportion of the poorly described languages in the sample. The previous results might be the artifacts of the data sparseness in WALS and the application of the low cutoff for the minimum feature coverage.

Another advantage of our approach is the use of the two complexity metrics on a comprehensive global sample. Previous empirical studies either focused on one grammatical domain, such as the number of cases (*20*) or verbal synthesis (*21*), or they included a wide variety of features corresponding to different coding procedures and interpretations of complexity [see studies based on WALS (*23*), such as (*6*, *22*, *26*)]. Moreover, we followed a systematic focused approach toward delineating fusion and informativity, which allows us to make principled decisions about the choice of the Grambank features for each metric and avoid including features that did not align with either of the described interpretations. Further studies could explore whether other grammatical complexity dimensions are sensitive to the influence of sociodemographic factors. One promising avenue for future research would be measuring the degree to which languages deviate from the principle of “one-meaning–one-form” (*25*) on a cross-linguistic scale.

All in all, the previous positive findings might be artifacts of small nonrepresentative samples, metrics subsuming grammatical features falling under distinct complexity dimensions, or methods not sufficiently controlling for genealogy and geography. Having overcome these past limitations and used a large sample offering greater statistical power, we find no evidence that societies of strangers speak grammatically less complex languages and that sociodemographic factors used in this study are strong drivers of fusion and informativity. Future studies could build on the methodological approach adopted in this paper and examine the impact of a more nuanced set of sociohistorical variables. The global Grambank dataset could be used to study other ambitious questions about linguistic diversity and language evolution.

While other language structures [e.g., lexicon (*5*, *50*, *51*)] or individual grammatical features, e.g., case marking (*20*, *42*), might adapt to changing sociodemographic factors, we find no evidence that the two dimensions of grammatical complexity measured here respond to sociolinguistic pressures in the hypothesized manner. We find that phylogenetic inheritance and borrowing between near neighbors explain most of the distribution of grammatical complexity among the world’s languages with respect to these two variables. This finding suggests that, even if speaker population size does play a role in driving down language complexity, the strength of selection is weak. Population size can change rapidly and unpredictably due to external events (wars, diseases, and migrations), let alone natural population growth (*52*). These changes might not leave noticeable traces on grammatical complexity for two reasons: Either the phylogenetically stable nature of these complexity variables constrains the rate at which these traits can adapt to their sociolinguistic environment, or the lability of population size means adaptation lags behind the selection pressures from the new selective regime.

Future work should explore the potential effects of other sociodemographic factors that are more fine-grained than the currently available demographic variables. Attention should be devoted to those factors that change at a relatively slow pace and hence provide relatively enduring selection pressures. Future studies should carefully consider the interplay between genealogy and geography when modeling the adaptation of languages to their environments and explain why some features might be more sensitive than others to sociolinguistic pressures.

## MATERIALS AND METHODS

### Datasets

#### Metrics

In this study, we want to focus on two delineated interpretations of grammatical complexity to better understand the forces shaping them:

1) the degree of fusion [the extent to which the language relies on phonologically fused markers (*53*)] and

2) the number of explicit and obligatory grammatical distinctions not routinely marked in all languages.

These linguistic phenomena are estimated by two metrics which are based on features available in Grambank v1.0 (https://doi.org/10.5281/zenodo.7740140). The first metric measures fusion. It accounts for phonologically fused grammatical markers, with scores increasing for having more phonologically fused markers. For each Grambank feature that concerns fused marking, a language can get 1 “fusion score” if it is coded as “present” in the database. If for a given feature, the language does not use the fused marker (is coded as “absent”), then it receives 0 fusion scores. We then take the mean of all these fusion scores per language to construct the score.

For instance, for plural marking on nouns, a language like English that forms plural forms with phonologically fused markers (*-s*) gets a 1 fusion score for this feature. By contrast, languages like Rapa Nui or Māori that lack phonologically fused markers for the purpose of nominal plural marking, or Vietnamese with no plural marking on nouns, are assigned 0 for this fusion feature. Overall, this metric systematically targets phonologically fused grammatical markers and can be compared to other metrics that capture the degree of morphological complexity (*6*), inventory complexity (*54*), and syntheticity (*16*).

We do not include features associated with derivation (GB047, GB048, and GB049) or morphosyntactic marking (GB146). In addition, we exclude the features related to the morphological case on pronouns (GB071 and GB073) as in languages where case marking is present, pronouns, as opposed to nouns, are especially prone to suppletion, which represents an instance of nonlinear rather than additive morphology.

The fusion metric is built on Grambank data, which, in turn, relies on reading grammatical descriptions and communication with experts. We acknowledge that different authors may have different approaches to what they define as “fused” and “phonologically independent.” However, despite these potential differences, the resulting fusion scores are in agreement with how previous works rank the world’s languages in their morphological complexity (see Materials and Methods for the comparison of fusion scores and the scores and judgments of morphological complexity in previous literature).

The second metric consists of features that contribute to explicit obligatory marking of grammatical and semantic distinctions that are not routinely marked in all languages. To ensure this, we exclude the typically overt grammatical domains related to negation, possession, comparative constructions, polar interrogation, and marking of A and P arguments (with the help of word order, case on nominal words, or indexes on verbs). Languages get scores for the presence of informativity features. Phonologically fused and independent markers contribute equally to the final informativity score when they address the same grammatical function. This means that if a language has an obligatory marking of plurality with phonologically fused and/or phonologically independent markers, it will get assigned 1 informativity score. Using the previous example, English, Māori, and Rapa Nui would all be assigned 1, while Vietnamese would have 0. This metric reveals the degree of grammatical marking in languages and is similar in spirit to measuring grammaticity (*16*) with a caveat that we exclude certain domains that are usually explicitly and obligatorily marked in most languages (negation, polar interrogation, etc.).

Next, the scores for each grammatical function in both metrics are summed, and the mean value is obtained for each language, so that the possible minimum for a language that has no fusion-related or no informative features is 0, and the possible maximum value is 1 if a language has all features that count as fusion-related or informative. In reality, we find no languages that reach the maximum scores on either of the metrics, but various languages (almost) approach the minimum of 0. For instance, one of the lowest-scoring (0) languages on fusion is Hu (Austroasiatic), lacking all fusion features, while the lowest informativity score of 0.1 is obtained by Jukun-Takum (Atlantic-Congo). Tariana (Arawakan) reaches the highest scores in both metrics: 0.7 on fusion and 0.66 on informativity. Next, the scores ranging between 0 and 1 are standardized to the mean of 0 and the variance of 1.

We measure fusion and informativity only for those languages that are well-described in Grambank (*28*) and remove languages with more than 25% of missing values across all Grambank features. Out of 2430 languages in the dataset, we compute the fusion and informativity scores for 1291 languages. This way, the resulting scores are robust representations of the targeted complexity dimensions.

Despite our focus on phonologically fused markers rather than morphological inflections, our metric of fusion is still comparable with what other studies measured as morphological complexity. For instance, according to the metric based on a variety of WALS features, Turkish ranked extremely high (0.775) and Vietnamese (Austroasiatic) was the lowest-scoring language (0.141) (*26*). In our data, we do not quantify fusion scores for Vietnamese but observe a divide between Turkish and some Austroasiatic languages lacking all fusion features: Turkish is assigned 0.44 with the maximally bound language Tariana (Arawakan) reaching as high as 0.7, while the fusion score of Thavung (same Vietic branch as Vietnamese), Hu, Prai, and Rumai Palaung is equal 0. Similarly, our metric captures the contrasts between languages from Kiranti and Kuki-Chin branches as suggested in (*19*). Camling (Sino-Tibetan) is claimed to show extreme morphological complexity and scores 0.42 in our metric, while Mara Chin (Sino-Tibetan) is said to have lost complexity, which is reflected in its lower score: 0.12 (*19*). Furthermore, the geographical patterns in the distribution of fusion are in line with typological literature and complexity studies. One prominent hotspot of low fusion is located in mainland Southeast Asia, which is expected on the basis of the typological profiles of languages in this area (*55*), and another one is in West Africa, which is in line with the proposal of the low-complexity belt (*56*).

#### Sociodemographic variables

Fusion and informativity are predicted on the basis of the following demographic and social variables: number of L1 speakers and and Vehicularity (along with the proportion of L2 speakers on the sample of 120 languages) (*57*) and number of linguistic neighbors, the status of the language (official/not official), and usage in education (language of education/not language of education), obtained from supplementary information available in (*41*), with data gathered by the authors or originally retrieved from World Language Mapping System v16 and v17 (WLMS, http://worldgeodatasets.com) and (*58*).

We log-transform the raw numbers of L1 speakers with a base of 10 and then standardize this variable along with the number of linguistic neighbors to have a mean of 0 and variance of 1. Standardization is done to eliminate the potential effects of extreme outliers on the results of the spatiophylogenetic modeling. The log-transformed (but not standardized) number of L1 speakers is used for implementing the interaction term between this variable and Vehicularity (or the L2 proportion on the sample of 120 languages).

The number of linguistic neighbors was calculated as the number of intersections between a 10,000 km^2^ circle of a given language and the polygons of other languages (*41*). The polygons and single-point locations were obtained from WLMS v16 and v17; single-point locations were used to approximate language areas using Voronoi projections where WLMS provided no polygons (*41*).

The status of the language is a binary variable: The language status is either not official or official at the national level, and the language is either used or not used in education. The languages are coded as official if they are formally recognized as such or if they are treated as the main languages of education, commerce, media, and government in countries that do not formally recognize any language as official, such as Australia (*41*). In these cases, these languages are also coded as languages of education if no other language serves as the language of education in the territory (*41*). All minority languages that were not recognized as official but were used as media of instruction in education according to *L’aménagement linguistique dans le monde* (*58*) were also assigned to be languages of education [see (*41*) and accompanying materials for more details on coding of the used sociodemographic variables].

We acknowledge that the modeling of grammatical features with fluctuating sociodemographic predictors poses challenges. We note that (i) the data from Grambank and the sources of sociodemographic variables are typical of contemporary populations and (ii) the sociodemographic variables are particularly prone to change more rapidly (*33*, *59*). Previous studies interpret the correlation between synchronic grammatical and sociodemographic structures diachronically. However, it is not clear how much time is necessary for the changes in the sociolinguistic environment to set in motion the changes to grammatical structures. Moreover, the use of common demographic variables, such as population size, the proportion of L2 speakers, and the number of linguistic neighbors, as proxies for exotericity has been questioned in a number of studies (*21*, *34*, *60*, *61*). While these limitations are inherent to all cross-linguistic studies on the link between grammatical structures and exotericity, our study overcomes past limitations concerning the sample size, control for sources of nonindependence, and the choice of linguistic features.

#### Phylogenetic and geographic information

We use Glottolog 4.5 (*43*) for information on the locations and language family of the languages in our sample. The AUTOTYP database (*38*) provided information on the distribution of languages across 24 language areas.

To model the evolutionary changes in these features over time and control for shared ancestry, we map grammatical complexity scores and values of sociodemographic variables onto the global EDGE phylogeny (*44*). This global supertree integrates language classification information from Glottolog and published phylogenies, as well as their locations.

### Spatiophylogenetic modeling

We adopt a spatiophylogenetic modeling technique pioneered by (*45*). This Bayesian approach uses an integrated nested Laplace approximation (INLA) (*62*, *63*) to estimate the joint posterior distribution of model parameters and is implemented in R (*64*) package INLA (*62*). On top of evaluating fixed effects coefficients, spatiophylogenetic modeling allows us to calculate the relative influence of random effects—here, spatial (geographic distances) and phylogenetic relationships—on the response variable.

#### Building random effects

To incorporate phylogenetic and spatial relationships as random effects in the models, we undertake several steps to represent these relationships in the form of precision matrices as required within INLA. We make these matrices comparable by standardizing them to have a variance of 1. This is done in the following way.

We build a phylogenetic variance-covariance matrix that quantifies the shared branch lengths between languages on the global tree with the assumption of a Brownian motion model of evolution using the ‘vcv.phylo’ function in ape (*65*). The tree serves to build a standardized phylogenetic precision matrix by applying the ‘inverseA’ function in the MCMCglmm package (*66*).

We follow a similar set of steps to calculate and standardize spatial matrices. First, we calculate a variance-covariance matrix under the Matérn spatial covariance function implemented in the ‘varcov.spatial’ function in geoR package (*67*). Second, we standardize the variance-covariance matrix by the variance and invert it to create a precision matrix. Last, the standardized variance-covariance matrix is transformed into the precision matrix. We estimate two spatial matrices for two sets of parameters: (i) ϕ = 1.25 and κ = 1 (“local set”) and (ii) ϕ = 17 and κ = 1 (“regional set”) [see (*68*) and (*69*) for other examples of how this type of control for spatial autocorrelation is implemented]. These parameters of the spatial covariance matrix were chosen to differentiate between two assumptions: Under parameters corresponding to the local set, the diffusion of similar metric scores between languages is not likely across distances more than 1000 km, while with the regional set parameters, the diffusion can take place over several thousands of kilometers (see fig. S1 for details). Fitting the models with each of these matrices allows us to compare which assumption about the diffusion of metrics scores corresponds to our data: are languages more likely to have similar scores only locally across hundreds of kilometers or is diffusion likely across larger regions, such as continents or large language areas spanning thousands of kilometers? For comparison, we also introduce the third control for spatial autocorrelation: random effects of 24 areas from the AUTOTYP database (*38*), where each area is treated as independent from each other, whereby we neglect geographic distances between the areas. These three different spatial effects are seen in competition with each other and represent different ways of operationalizing the influence of contact between neighboring languages. The models incorporating phylogenetic effects and local versions of spatial effects predict fusion and informativity best (see Tables 1 and 2).

#### Sensitivity testing

We use penalized complexity (PC) priors (*70*) on the precision of the likelihood and phylogenetic and spatial effects. Precision matrices are standardized to have a variance of 1. In the main text results, PC priors are set so that 10% of the prior probability density of the SD of the likelihood or random effects fall above 1. As a sensitivity test, we vary the probability density at 1, 10, 50, and 99%, but this does not affect our conclusions (see table S5).

#### Measuring phylogenetic signal

We estimate Pagel’s lambda (λ) (*48*) to measure the phylogenetic signal of fusion and informativity on the global EDGE tree using R package, phytools (*71*). The values of λ typically range from 0 to 1. λ = 1 implies a high phylogenetic signal, which means that the scores evolve in a manner expected under a Brownian motion model. Conversely, λ = 0 suggests no phylogenetic signal and indicates that the distribution of scores evolved independently from the phylogenetic relationships between the languages in the phylogeny.
