# Supplementary material for: Societies of strangers do not speak less complex languages
Source: Sci Adv. 2023 Aug 16;9(33):eadf7704. doi: 10.1126/sciadv.adf7704 (PMC10431698; doi:10.1126/sciadv.adf7704)
Supplement: Supplementary file 1 — Fig. S1 Tables S1 to S5 [file sciadv.adf7704_sm.pdf]

CORRECTED 19 JANUARY 2024; SEE ERRATUM

Science Advances
AAAS

Supplementary Materials for

Societies of strangers do not speak less complex languages

Olena Shcherbakova *et al.*

Corresponding author: Olena Shcherbakova, olena_shcherbakova@eva.mpg.de

Sci. Adv. **9**, eadf7704 (2023)
DOI: 10.1126/sciadv.adf7704

This PDF file includes:

Fig. S1
Tables S1 to S5

Table S1. The overview of Grambank features in metrics of fusion and informativity. The score of "1" is assigned to the fusion features if they are present in respective languages. Features that receive "0" are not involved in the fusion metric. Informativity metric assigns the score of "1" for the presence of at least one feature within the labelled group of features. The features that do not contribute to the informativity metric are indicated as "0".

**Feature_ID**	**Feature**	**fusion**	**informativity**
GB074	Are there prepositions?	0	0
GB075	Are there postpositions?	0	0
GB303	Is there a phonologically free antipassive marker ("particle" or "auxiliary")?	0	antipassive
GB520	Can aspect be marked by a non-inflecting word ("auxiliary particle")?	0	aspect
GB519	Can mood be marked by a non-inflecting word ("auxiliary particle")?	0	mood
GB299	Can standard negation be marked by a non-inflecting word ("auxiliary particle")?	0	0
GB317	Is dual number regularly marked in the noun phrase by a phonologically free element?	0	dual
GB320	Is paucal number regularly marked in the noun phrase by a phonologically free element?	0	paucal
GB318	Is plural number regularly marked in the noun phrase by a phonologically free element?	0	plural
GB316	Is singular number regularly marked in the noun phrase by a phonologically free element?	0	singular
GB319	Is trial number regularly marked in the noun phrase by a phonologically free element?	0	trial

GB302	Is there a phonologically free passive marker ("particle" or "auxiliary")?	0	passive
GB262	Is there a clause-initial polar interrogative particle?	0	0
GB263	Is there a clause-final polar interrogative particle?	0	0
GB264	Is there a polar interrogative particle that most commonly occurs neither clause-initially nor clause-finally?	0	0
GB521	Can tense be marked by a non-inflecting word ("auxiliary particle")?	0	tense
GB071	Are there morphological cases for independent personal pronominal core arguments (i.e. S/A/P)?	0	0
GB146	Is there a morpho-syntactic distinction between predicates expressing controlled versus uncontrolled events or states?	0	control
GB047	Is there a productive morphological pattern for deriving an action/state noun from a verb?	0	0
GB048	Is there a productive morphological pattern for deriving an agent noun from a verb?	0	0
GB049	Is there a productive morphological pattern for deriving an object noun from a verb?	0	0
GB073	Are there morphological cases for independent oblique personal pronominal arguments (i.e. not S/A/P)?	0	0
GB148	Is there a morphological antipassive marked on the lexical verb?	1	antipassive

GB070	Are there morphological cases for non-pronominal core arguments (i.e. S/A/P)?	1	0
GB091	Can the A argument be indexed by a suffix/enclitic on the verb in the simple independent clause?	1	0
GB092	Can the A argument be indexed by a prefix/proclitic on the verb in the simple independent clause?	1	0
GB093	Can the P argument be indexed by a suffix/enclitic on the verb in the simple independent clause?	1	0
GB094	Can the P argument be indexed by a prefix/proclitic on the verb in the simple independent clause?	1	0
GB086	Is a morphological distinction between perfective and imperfective aspect available on verbs?	1	aspect
GB120	Can aspect be marked by an inflecting word ("auxiliary verb")?	1	aspect
GB188	Is there any productive augmentative marking on the noun (exclude marking by system of nominal classification only)?	1	augmentative
GB275	Is there a bound comparative degree marker on the property word in a comparative construction?	1	0
GB187	Is there any productive diminutive marking on the noun (exclude marking by system of nominal classification only)?	1	diminutive
GB170	Can an adnominal property word agree with the noun in gender/noun class?	1	0

GB172	Can an article agree with the noun in gender/noun class?	1	0
GB198	Can an adnominal numeral agree with the noun in gender/noun class?	1	0
GB119	Can mood be marked by an inflecting word ("auxiliary verb")?	1	mood
GB312	Is there overt morphological marking on the verb dedicated to mood?	1	mood
GB107	Can standard negation be marked by an affix, clitic or modification of the verb?	1	0
GB298	Can standard negation be marked by an inflecting word ("auxiliary verb")?	1	0
GB171	Can an adnominal demonstrative agree with the noun in gender/noun class?	1	0
GB184	Can an adnominal property word agree with the noun in number?	1	0
GB185	Can an adnominal demonstrative agree with the noun in number?	1	0
GB186	Can an article agree with the noun in number?	1	0
GB043	Is there productive morphological dual marking on nouns?	1	dual
GB166	Is there productive morphological paucal marking on nouns?	1	paucal
GB044	Is there productive morphological plural marking on nouns?	1	plural
GB042	Is there productive overt morphological singular marking on nouns?	1	singular

GB165	Is there productive morphological trial marking on nouns?	1	trial
GB147	Is there a morphological passive marked on the lexical verb?	1	passive
GB285	Can polar interrogation be marked by a question particle and verbal morphology?	1	0
GB286	Can polar interrogation be indicated by overt verbal morphology only?	1	0
GB430	Can adnominal possession be marked by a prefix on the possessor?	1	0
GB431	Can adnominal possession be marked by a prefix on the possessed noun?	1	0
GB432	Can adnominal possession be marked by a suffix on the possessor?	1	0
GB433	Can adnominal possession be marked by a suffix on the possessed noun?	1	0
GB115	Is there a phonologically bound reciprocal marker on the verb?	1	reciprocity
GB114	Is there a phonologically bound reflexive marker on the verb?	1	reflexivity
GB121	Can tense be marked by an inflecting word ("auxiliary verb")?	1	tense
GB082	Is there overt morphological marking of present tense on verbs?	1	tense
GB083	Is there overt morphological marking on the verb dedicated to past tense?	1	tense
GB084	Is there overt morphological marking on the verb dedicated to future tense?	1	tense

GB103	Is there a benefactive applicative marker on the verb (including indexing)?	1	benefactive
GB108	Is there directional or locative morphological marking on verbs?	1	directional
GB104	Is there an instrumental applicative marker on the verb (including indexing)?	1	instrumental
GB149	Is there a morphologically marked inverse on verbs?	1	inverse
GB152	Is there a morphologically marked distinction between simultaneous and sequential clauses?	1	simultanseq
GB151	Is there an overt verb marker dedicated to signalling coreference or noncoreference between the subject of one clause and an argument of an adjacent clause ("switch reference")?	1	switch reference
GB072	Are there morphological cases for oblique non-pronominal NPs (i.e. not S/A/P)?	1	0
GB079	Do verbs have prefixes/proclitics, other than those that only mark A, S or P (do include portmanteau: A & S + TAM)?	1	0
GB080	Do verbs have suffixes/enclitics, other than those that only mark A, S or P (do include portmanteau: A & S + TAM)?	1	0
GB081	Is there productive infixation in verbs?	1	0
GB089	Can the S argument be indexed by a suffix/enclitic on the verb in the simple independent clause?	1	0
GB090	Can the S argument be indexed by a prefix/proclitic on the verb in the simple independent clause?	1	0

GB113	Are there verbal affixes or clitics that turn intransitive verbs into transitive ones?	1	0
GB155	Are causatives formed by affixes or clitics on verbs?	1	0
GB177	Can the verb carry a marker of animacy of argument, unrelated to any gender/noun class of the argument visible in the NP domain?	1	argumentanimacy
GB059	Is the adnominal possessive construction different for alienable and inalienable nouns?	0	alienability
GB057	Are there numeral classifiers?	0	numera classifers
GB058	Are there possessive classifiers?	0	possessive classifiers
GB333	Is there a decimal numeral system?	0	0
GB334	Is there synchronic evidence for any element of a quinary numeral system?	0	0
GB335	Is there synchronic evidence for any element of a vigesimal numeral system?	0	0
GB336	Is there a body-part tallying system?	0	0
GB022	Are there prenominal articles?	0	0
GB023	Are there postnominal articles?	0	0
GB314	Can augmentative meaning be expressed productively by a shift of gender/noun class?	0	augmentative
GB276	Is there a non-bound comparative degree marker modifying the property word in a comparative construction?	0	0

GB265	Is there a comparative construction that includes a form that elsewhere means 'surpass, exceed'?	0	0
GB266	Is there a comparative construction that employs a marker of the standard which elsewhere has a locational meaning?	0	0
GB270	Can comparatives be expressed using two conjoined clauses?	0	0
GB273	Is there a comparative construction with a standard marker that elsewhere has neither a locational meaning nor a 'surpass/exceed' meaning?	0	0
GB421	Is there a preposed complementizer in complements of verbs of thinking and/or knowing?	0	0
GB422	Is there a postposed complementizer in complements of verbs of thinking and/or knowing?	0	0
GB315	Can diminutive meaning be expressed productively by a shift of gender/noun class?	0	diminutive
GB031	Is there a dual or unit augmented form (in addition to plural or augmented) for all person categories in the pronoun system?	0	pronoundualaug
GB136	Is the order of core argument (i.e. S/A/P) constituents fixed?	0	0
GB408	Is there any accusative alignment of flagging?	0	0
GB409	Is there any ergative alignment of flagging?	0	0
GB410	Is there any neutral alignment of flagging?	0	0

GB053	Is there a gender/noun class system where animacy is a factor in class assignment?	0	genderanimacy
GB054	Is there a gender/noun class system where plant status is a factor in class assignment?	0	genderplant
GB051	Is there a gender/noun class system where sex is a factor in class assignment?	0	gendersex
GB052	Is there a gender/noun class system where shape is a factor in class assignment?	0	gendershape
GB197	Is there a male/female distinction in 1st person independent pronouns?	0	pronoungender1
GB196	Is there a male/female distinction in 2nd person independent pronouns?	0	pronoungender2
GB030	Is there a gender distinction in independent 3rd person pronouns?	0	pronoungender3
GB137	Can standard negation be marked clause-finally?	0	0
GB138	Can standard negation be marked clause-initially?	0	0
GB039	Is there nonphonological allomorphy of noun number markers?	0	0
GB041	Are there several nouns (more than three) which are suppletive for number?	0	0
GB025	What is the order of adnominal demonstrative and noun?	0	0
GB024	What is the order of numeral and noun in the NP?	0	0

GB065	What is the pragmatically unmarked order of adnominal possessor noun and possessed noun?	0	0
GB193	What is the order of adnominal property word and noun?	0	0
GB203	What is the order of the adnominal collective universal quantifier ('all') and the noun?	0	0
GB130	What is the pragmatically unmarked order of S and V in intransitive clauses?	0	0
GB131	Is a pragmatically unmarked constituent order verb-initial for transitive clauses?	0	0
GB132	Is a pragmatically unmarked constituent order verb-medial for transitive clauses?	0	0
GB133	Is a pragmatically unmarked constituent order verb-final for transitive clauses?	0	0
GB257	Can polar interrogation be marked by intonation only?	0	0
GB260	Can polar interrogation be indicated by a special word order?	0	0
GB291	Can polar interrogation be marked by tone?	0	0
GB297	Can polar interrogation be indicated by a V-not-V construction?	0	0
GB250	Can predicative possession be expressed with a transitive 'habeo' verb?	0	0
GB252	Can predicative possession be expressed with an S-like possessum and a locative-coded possessor?	0	0

GB253	Can predicative possession be expressed with an S-like possessum and a dative-coded possessor?	0	0
GB254	Can predicative possession be expressed with an S-like possessum and a possessor that is coded like an adnominal possessor?	0	0
GB256	Can predicative possession be expressed with an S-like possessor and a possessum that is coded like a comitative argument?	0	0
GB026	Can adnominal property words occur discontinuously?	0	0
GB306	Is there a phonologically independent non-bipartite reciprocal pronoun?	0	reciprocity
GB305	Is there a phonologically independent reflexive pronoun?	0	reflexivity
GB327	Can the relative clause follow the noun?	0	0
GB328	Can the relative clause precede the noun?	0	0
GB046	Is there an associative plural marker for nouns?	0	assocplural
GB028	Is there a distinction between inclusive and exclusive?	0	clusivity
GB027	Are nominal conjunction and comitative expressed by different elements?	0	comitative
GB117	Is there a copula for predicate nominals?	0	copulaprednom
GB325	Is there a count/mass distinction in interrogative quantifiers?	0	count_mass
GB020	Are there definite or specific articles?	0	definitearticles

GB035	Are there three or more distance contrasts in demonstratives?	0	demonstartivedistance
GB038	Are there demonstrative classifiers?	0	demonstrative classifiers
GB036	Do demonstratives show an elevation distinction?	0	demonstrativeelevation
GB037	Do demonstratives show a visible-nonvisible distinction?	0	demonstrativevisibility
GB140	Is verbal predication marked by the same negator as all of the following types of predication: locational, existential and nominal?	0	differentneg
GB322	Is there grammatical marking of direct evidence (perceived with the senses)?	0	evidentiality_direct
GB323	Is there grammatical marking of indirect evidence (hearsay, inference, etc.)?	0	evidentiality_indirect
GB126	Is there an existential verb?	0	existentialverb
GB021	Do indefinite nominals commonly have indefinite articles?	0	indef
GB309	Are there multiple past or multiple future tenses, distinguishing distance from Time of Reference?	0	multipletense
GB415	Is there a politeness distinction in 2nd person forms?	0	politeness
GB127	Are different posture verbs used obligatorily depending on an inanimate locatum's shape or position (e.g. 'to lie' vs. 'to stand')?	0	postureverbs
GB139	Is there a difference between imperative (prohibitive) and declarative negation constructions?	0	prohibitive

GB167	Is there a logophoric pronoun?	0	pronounlog
GB116	Do verbs classify the shape, size or consistency of absolutive arguments by means of incorporated nouns, verbal affixes or suppletive verb stems?	0	verbclassify
GB068	Do core adjectives (defined semantically as property concepts such as value, shape, age, dimension) act like verbs in predicative position?	0	0
GB069	Do core adjectives (defined semantically as property concepts; value, shape, age, dimension) used attributively require the same morphological treatment as verbs?	0	0
GB095	Are variations in marking strategies of core participants based on TAM distinctions?	0	0
GB096	Are variations in marking strategies of core participants based on verb classes?	0	0
GB098	Are variations in marking strategies of core participants based on person distinctions?	0	0
GB099	Can verb stems alter according to the person of a core participant?	0	0
GB105	Can the recipient in a ditransitive construction be marked like the monotransitive patient?	0	0
GB109	Is there verb suppletion for participant number?	0	0
GB110	Is there verb suppletion for tense or aspect?	0	0
GB111	Are there conjugation classes?	0	0

GB118	Are there serial verb constructions?	0	0
GB122	Is verb compounding a regular process?	0	0
GB123	Are there verb-adjunct (aka light-verb) constructions?	0	0
GB124	Is incorporation of nouns into verbs a productive intransitivizing process?	0	0
GB129	Is there a notably small number, i.e. about 100 or less, of verb roots in the language?	0	0
GB134	Is the order of constituents the same in main and subordinate clauses?	0	0
GB135	Do clausal objects usually occur in the same position as nominal objects?	0	0
GB150	Is there clause chaining?	0	0
GB156	Is there a causative construction involving an element that is unmistakably grammaticalized from a verb for 'to say'?	0	0
GB158	Are verbs reduplicated?	0	0
GB159	Are nouns reduplicated?	0	0
GB160	Are elements apart from verbs or nouns reduplicated?	0	0
GB192	Is there a gender system where a noun's phonological properties are a factor in class assignment?	0	0
GB204	Do collective ('all') and distributive ('every') universal quantifiers differ in their forms or their syntactic positions?	0	0

GB296	Is there a phonologically or morphosyntactically definable class of ideophones that includes ideophones depicting imagery beyond sound?	0	0
GB300	Does the verb for 'give' have suppletive verb forms?	0	0
GB301	Is there an inclusory construction?	0	0
GB304	Can the agent be expressed overtly in a passive clause?	0	0
GB313	Are there special adnominal possessive pronouns that are not formed by an otherwise regular process?	0	0
GB321	Is there a large class of nouns whose gender/noun class is not phonologically or semantically predictable?	0	0
GB324	Is there an interrogative verb for content interrogatives (who?, what?, etc.)?	0	0
GB326	Do (nominal) content interrogatives normally or frequently occur in situ?	0	0
GB329	Are there internally-headed relative clauses?	0	0
GB330	Are there correlative relative clauses?	0	0
GB331	Are there adjoined relative clauses?	0	0
GB400	Are all person categories neutralized in some voice, tense, aspect, mood and/or negation?	0	0
GB401	Is there a class of patient-labile verbs?	0	0

GB402	Does the verb for 'see' have suppletive verb forms?	0	0
GB403	Does the verb for 'come' have suppletive verb forms?	0	0
GB522	Can the S or A argument be omitted from a pragmatically unmarked clause when the referent is inferrable from context ("pro-drop" or "null anaphora")?	0	0

Table S2. WAIC values and quantiles (0.025, 0.5, and 0.975) of estimates of models predicting fusion and informativity (including the nonlinear implementations of L1 speakers): with random effects, with random and fixed effects, and with fixed effects. The sample column indicates whether the analyses were done on the entire sample ("entire") of 1,291 languages or the sample of 120 languages for which the numbers of L2 speakers were available ("L2").

model	response	effect	2.5%	50%	97.5 %	WAIC	sample
Phylogenetic+Spatial: local	fusion	phylogenetic SD	1.51	1.73	2.00	1,821.88	entire
		spatial SD	0.28	0.34	0.41	1,821.88	entire
		Intercept	-0.02	0.00	0.02	1,821.88	entire
Phylogenetic		phylogenetic SD	1.99	2.19	2.42	1,941.21	entire
		Intercept	-0.03	0.00	0.03	1,941.21	entire
Phylogenetic+Areal		phylogenetic SD	1.79	2.01	2.26	2,015.61	entire
		areal SD	0.09	0.16	0.28	2,015.61	entire
		Intercept	-0.06	0.03	0.13	2,015.61	entire
Phylogenetic+Spatial: regional		phylogenetic SD	1.23	1.47	1.75	2,056.58	entire
		spatial SD	0.47	0.61	0.79	2,056.58	entire
		Intercept	-0.03	0.00	0.03	2,056.58	entire
Spatial: local		spatial SD	0.74	0.80	0.85	2,259.25	entire
		Intercept	-0.02	0.00	0.03	2,259.25	entire
Spatial: regional		spatial SD	0.87	1.03	1.21	2,423.66	entire
		Intercept	-0.03	0.00	0.03	2,423.66	entire
Areal		areal SD	0.44	0.58	0.80	2,866.67	entire

		Intercept	0.08	0.32	0.55	2,866.67	entire
Phylogenetic+Spatial: local+L1 speakers (linear)		phylogenetic SD	1.53	1.75	2.02	1,796.34	entire
		spatial SD	0.28	0.34	0.41	1,796.34	entire
		Intercept	-0.02	0.00	0.02	1,796.34	entire
		L1	0.03	0.07	0.12	1,796.34	entire
Phylogenetic+Spatial: local+L1 speakers (nonlinear)		phylogenetic SD	1.53	1.75	2.02	1,797.20	entire
		spatial SD	0.28	0.34	0.41	1,797.20	entire
		Intercept	-0.02	0.00	0.03	1,797.20	entire
		social SD: L1	0.00	0.01	0.03	1,797.20	entire
Phylogenetic+Spatial: local+L1 speakers (linear)+Vehicularity		phylogenetic SD	1.53	1.75	2.02	1,798.71	entire
		spatial SD	0.27	0.34	0.40	1,798.71	entire
		Intercept	-0.04	-0.01	0.02	1,798.71	entire
		L1	0.01	0.06	0.11	1,798.71	entire
		Vehicularity	-0.03	0.09	0.21	1,798.71	entire
Phylogenetic+Spatial: local+L1_log10:Vehicularity		phylogenetic SD	1.52	1.74	2.01	1,814.65	entire
		spatial SD	0.27	0.34	0.40	1,814.65	entire
		Intercept	-0.04	-0.01	0.01	1,814.65	entire
		L1*Vehicularity	0.01	0.02	0.04	1,814.65	entire

Phylogenetic+Spatial: local+Vehicularity		phylogenetic SD	1.52	1.74	2.01	1,814.82	entire
		spatial SD	0.27	0.34	0.40	1,814.82	entire
		Intercept	-0.04	-0.01	0.01	1,814.82	entire
		Vehicularity	0.03	0.14	0.26	1,814.82	entire
Phylogenetic+Spatial: local+Neighbours		phylogenetic SD	1.52	1.74	2.01	1,818.78	entire
		spatial SD	0.27	0.34	0.41	1,818.78	entire
		Intercept	-0.03	0.00	0.02	1,818.78	entire
		Neighbours	-0.01	0.02	0.05	1,818.78	entire
Phylogenetic+Spatial: local+Official		phylogenetic SD	1.50	1.72	1.99	1,820.45	entire
		spatial SD	0.28	0.34	0.41	1,820.45	entire
		Intercept	-0.03	-0.01	0.02	1,820.45	entire
		Official status	0.01	0.17	0.34	1,820.45	entire
Phylogenetic+Spatial: local+Education		phylogenetic SD	1.50	1.72	1.99	1,821.36	entire
		spatial SD	0.28	0.34	0.41	1,821.36	entire
		Intercept	-0.04	-0.01	0.01	1,821.36	entire
		Education	0.01	0.15	0.29	1,821.36	entire
L1 speakers (nonlinear)		Intercept	0.08	0.17	0.25	3,609.09	entire
		social SD: L1	0.06	0.14	0.47	3,609.09	entire

Education		Intercept	-0.09	-0.03	0.02	3,627.87	entire
		Education	0.26	0.46	0.66	3,627.87	entire
Official		Intercept	-0.08	-0.03	0.03	3,630.71	entire
		Official status	0.26	0.50	0.74	3,630.71	entire
L1_log10:Vehicularity		Intercept	-0.09	-0.03	0.02	3,634.12	entire
		L1*Vehicularity	0.03	0.05	0.08	3,634.12	entire
Vehicularity		Intercept	-0.09	-0.03	0.03	3,637.69	entire
		Vehicularity	0.11	0.29	0.46	3,637.69	entire
L1 speakers (linear)+Vehicularity		Intercept	-0.09	-0.03	0.03	3,639.72	entire
		L1	-0.07	0.00	0.06	3,639.72	entire
		Vehicularity	0.09	0.29	0.49	3,639.72	entire
L1 speakers (linear)		Intercept	-0.05	0.00	0.05	3,646.12	entire
		L1	-0.02	0.04	0.10	3,646.12	entire
Neighbours		Intercept	-0.05	0.00	0.06	3,648.08	entire
		Neighbours	-0.06	-0.01	0.03	3,648.08	entire
Phylogenetic+Spatial: local	informativity	phylogenetic SD	0.88	1.19	1.56	3,152.71	entire
		spatial SD	0.36	0.45	0.54	3,152.71	entire
		Intercept	-0.01	0.03	0.07	3,152.71	entire
Phylogenetic		phylogenetic SD	1.48	1.78	2.12	3,201.82	entire
		Intercept	-0.01	0.03	0.07	3,201.82	entire

Phylogenetic+Areal		phylogenetic SD	1.29	1.63	2.01	3,224.01	entire
		areal SD	0.05	0.14	0.29	3,224.01	entire
		Intercept	-0.04	0.05	0.16	3,224.01	entire
Phylogenetic+Spatial: regional		phylogenetic SD	0.60	0.90	1.38	3,225.33	entire
		spatial SD	0.49	0.65	0.85	3,225.33	entire
		Intercept	-0.01	0.03	0.08	3,225.33	entire
Spatial: local		spatial SD	0.58	0.65	0.72	3,302.19	entire
		Intercept	-0.01	0.03	0.07	3,302.19	entire
Spatial: regional		spatial SD	0.55	0.71	0.89	3,305.44	entire
		Intercept	-0.01	0.03	0.08	3,305.44	entire
Areal		areal SD	0.31	0.42	0.59	3,437.41	entire
		Intercept	0.00	0.18	0.37	3,437.41	entire
Phylogenetic+Spatial: local+L1 speakers (linear)+Vehicularity		phylogenetic SD	0.92	1.24	1.60	3,128.91	entire
		spatial SD	0.36	0.44	0.53	3,128.91	entire
		Intercept	-0.04	0.00	0.04	3,128.91	entire
		L1	-0.02	0.05	0.13	3,128.91	entire
		Vehicularity	0.12	0.31	0.49	3,128.91	entire
Phylogenetic+Spatial: local+L1 speakers (linear)		phylogenetic SD	0.97	1.28	1.66	3,132.76	entire
		spatial SD	0.35	0.43	0.53	3,132.76	entire

		Intercept	-0.01	0.03	0.07	3,132.76	entire
		L1	0.03	0.10	0.17	3,132.76	entire
Phylogenetic+Spatial: local+L1 speakers (nonlinear)		phylogenetic SD	0.96	1.27	1.65	3,135.03	entire
		spatial SD	0.35	0.43	0.53	3,135.03	entire
		Intercept	0.00	0.04	0.08	3,135.03	entire
		social SD: L1	0.00	0.01	0.03	3,135.03	entire
Phylogenetic+Spatial: local+L1_log10:Vehicularity		phylogenetic SD	0.89	1.20	1.57	3,135.58	entire
		spatial SD	0.36	0.44	0.54	3,135.58	entire
		Intercept	-0.05	0.00	0.04	3,135.58	entire
		L1*Vehicularity	0.03	0.06	0.09	3,135.58	entire
Phylogenetic+Spatial: local+Vehicularity		phylogenetic SD	0.87	1.18	1.55	3,136.39	entire
		spatial SD	0.36	0.44	0.54	3,136.39	entire
		Intercept	-0.05	0.00	0.04	3,136.39	entire
		Vehicularity	0.19	0.36	0.53	3,136.39	entire
Phylogenetic+Spatial: local+Neighbours		phylogenetic SD	0.88	1.20	1.57	3,153.06	entire
		spatial SD	0.36	0.44	0.54	3,153.06	entire
		Intercept	-0.01	0.03	0.07	3,153.06	entire
		Neighbours	-0.04	0.01	0.05	3,153.06	entire

Phylogenetic+Spatial: local+Education		phylogenetic SD	0.85	1.15	1.54	3,156.96	entire
		spatial SD	0.37	0.45	0.54	3,156.96	entire
		Intercept	-0.02	0.02	0.06	3,156.96	entire
		Education	-0.03	0.18	0.38	3,156.96	entire
Phylogenetic+Spatial: local+Official		phylogenetic SD	0.83	1.14	1.52	3,157.39	entire
		spatial SD	0.37	0.45	0.54	3,157.39	entire
		Intercept	-0.02	0.02	0.06	3,157.39	entire
		Official status	-0.03	0.21	0.45	3,157.39	entire
L1 speakers (linear)+Vehicularity		Intercept	-0.06	-0.01	0.05	3,642.42	entire
		L1	-0.15	-0.09	-0.02	3,642.42	entire
		Vehicularity	0.18	0.38	0.58	3,642.42	entire
Official		Intercept	-0.04	0.01	0.07	3,645.36	entire
		Official status	0.15	0.39	0.62	3,645.36	entire
Education		Intercept	-0.05	0.01	0.06	3,646.32	entire
		Education	0.11	0.31	0.51	3,646.32	entire
Vehicularity		Intercept	-0.05	0.01	0.06	3,647.35	entire
		Vehicularity	0.08	0.26	0.43	3,647.35	entire
L1_log10:Vehicularity		Intercept	-0.05	0.01	0.07	3,648.47	entire
		L1*Vehicularity	0.01	0.04	0.07	3,648.47	entire

Neighbours		Intercept	-0.02	0.04	0.09	3,649.32	entire
		Neighbours	-0.10	-0.06	-0.01	3,649.32	entire
L1 speakers (nonlinear)		Intercept	-0.02	0.04	0.12	3,652.78	entire
		social SD: L1	0.00	0.01	0.05	3,652.78	entire
L1 speakers (linear)		Intercept	-0.02	0.03	0.09	3,654.32	entire
		L1	-0.09	-0.03	0.03	3,654.32	entire
Phylogenetic+Spatial: local	fusion	phylogenetic SD	0.22	0.48	0.93	93.94	L2
		spatial SD	0.63	0.76	0.92	93.94	L2
		Intercept	0.33	0.38	0.44	93.94	L2
Spatial: local		spatial SD	0.68	0.81	0.98	141.91	L2
		Intercept	0.32	0.38	0.44	141.91	L2
Phylogenetic+Spatial: regional		phylogenetic SD	0.02	0.13	0.57	225.46	L2
		spatial SD	0.60	0.91	1.32	225.46	L2
		Intercept	0.29	0.38	0.48	225.46	L2
Spatial: regional		spatial SD	0.60	0.91	1.32	226.39	L2
		Intercept	0.29	0.38	0.48	226.39	L2
Areal		areal SD	0.33	0.53	0.86	249.95	L2
		Intercept	0.06	0.39	0.71	249.95	L2
Phylogenetic+Areal		phylogenetic SD	0.02	0.13	0.50	251.58	L2

		areal SD	0.31	0.52	0.85	251.58	L2
		Intercept	0.07	0.40	0.73	251.58	L2
Phylogenetic		phylogenetic SD	0.18	0.41	0.89	275.97	L2
		Intercept	0.25	0.38	0.51	275.97	L2
Phylogenetic+Spatial: local+L1 speakers (linear)		phylogenetic SD	0.22	0.51	1.00	93.25	L2
		spatial SD	0.63	0.75	0.91	93.25	L2
		Intercept	0.33	0.39	0.44	93.25	L2
		L1	-0.08	0.11	0.30	93.25	L2
Phylogenetic+Spatial: local+L1 speakers (nonlinear)		phylogenetic SD	0.21	0.49	0.99	93.87	L2
		spatial SD	0.63	0.76	0.91	93.87	L2
		Intercept	0.27	0.35	0.43	93.87	L2
		social SD: L1	0.00	0.01	0.03	93.87	L2
Phylogenetic+Spatial: local+L1_log10:L2 proportion (linear)		phylogenetic SD	0.22	0.48	0.93	94.18	L2
		spatial SD	0.64	0.77	0.93	94.18	L2
		Intercept	0.24	0.38	0.52	94.18	L2
		L1*L2 proportion	-0.13	0.01	0.14	94.18	L2
Phylogenetic+Spatial: local+L1 speakers		phylogenetic SD	0.20	0.48	0.97	96.52	L2

(nonlinear)+L2 proportion (nonlinear)		spatial SD	0.63	0.76	0.92	96.52	L2
		Intercept	0.14	0.31	0.48	96.52	L2
		social SD: L1	0.00	0.01	0.03	96.52	L2
		social SD: L2 proportion	0.00	0.01	0.03	96.52	L2
Phylogenetic+Spatial: local+L1 speakers (linear)+L2 proportion (linear)		phylogenetic SD	0.21	0.50	0.99	99.39	L2
		spatial SD	0.62	0.75	0.91	99.39	L2
		Intercept	0.29	0.42	0.56	99.39	L2
		L1	-0.09	0.10	0.29	99.39	L2
		L2 proportion	-0.87	-0.20	0.47	99.39	L2
Phylogenetic+Spatial: local+L2 proportion (linear)		phylogenetic SD	0.21	0.48	0.93	101.21	L2
		spatial SD	0.63	0.76	0.92	101.21	L2
		Intercept	0.30	0.43	0.57	101.21	L2
		L2 proportion	-0.92	-0.26	0.40	101.21	L2
Phylogenetic+Spatial: local+L2 proportion (nonlinear)		phylogenetic SD	0.21	0.47	0.93	101.40	L2
		spatial SD	0.63	0.76	0.92	101.40	L2
		Intercept	0.16	0.32	0.49	101.40	L2
		social SD: L2 proportion	0.00	0.01	0.03	101.40	L2
L1 speakers (nonlinear)		Intercept	0.18	0.33	0.48	284.00	L2

		social SD: L1	0.00	0.01	0.03	284.00	L2
L1 speakers (linear)		Intercept	0.25	0.39	0.53	284.20	L2
		L1	0.01	0.18	0.35	284.20	L2
L1 speakers (nonlinear)+L2 proportion (nonlinear)		Intercept	0.06	0.27	0.48	286.22	L2
		social SD: L1	0.00	0.01	0.03	286.22	L2
		social SD: L2 proportion	0.00	0.01	0.03	286.22	L2
L1 speakers (linear)+L2 proportion (linear)		Intercept	0.26	0.44	0.62	286.28	L2
		L1	0.00	0.17	0.35	286.28	L2
		L2 proportion	-0.91	-0.28	0.36	286.28	L2
L2 proportion (nonlinear)		Intercept	0.10	0.31	0.52	288.02	L2
		social SD: L2 proportion	0.00	0.01	0.03	288.02	L2
L2 proportion (linear)		Intercept	0.26	0.44	0.63	288.30	L2
		L2 proportion	-0.96	-0.32	0.32	288.30	L2
L1_log10:L2 proportion (linear)		Intercept	0.18	0.37	0.55	289.08	L2
		L1*L2 proportion	-0.11	0.02	0.14	289.08	L2
Phylogenetic+Spatial: local	informativity	phylogenetic SD	0.02	0.17	0.74	289.50	L2
		spatial SD	0.32	0.55	0.82	289.50	L2
		Intercept	0.01	0.13	0.25	289.50	L2

Spatial: local		spatial SD	0.31	0.53	0.79	295.04	L2
		Intercept	0.00	0.13	0.26	295.04	L2
Phylogenetic+Spatial: regional		phylogenetic SD	0.02	0.17	0.63	298.01	L2
		spatial SD	0.23	0.52	0.93	298.01	L2
		Intercept	-0.01	0.13	0.27	298.01	L2
Areal		areal SD	0.15	0.33	0.65	300.59	L2
		Intercept	-0.10	0.17	0.45	300.59	L2
Phylogenetic+Areal		phylogenetic SD	0.02	0.16	0.68	300.85	L2
		areal SD	0.17	0.36	0.68	300.85	L2
		Intercept	-0.11	0.18	0.49	300.85	L2
Spatial: regional		spatial SD	0.17	0.44	0.86	301.02	L2
		Intercept	-0.02	0.13	0.27	301.02	L2
Phylogenetic		phylogenetic SD	0.02	0.16	0.71	309.81	L2
		Intercept	-0.03	0.13	0.28	309.81	L2
Phylogenetic+Spatial: local+L1 speakers (linear)+L2 proportion (linear)		phylogenetic SD	0.02	0.17	0.67	288.06	L2
		spatial SD	0.35	0.58	0.86	288.06	L2
		Intercept	-0.03	0.16	0.35	288.06	L2
		L1	-0.18	0.05	0.28	288.06	L2
		L2 proportion	-0.98	-0.18	0.62	288.06	L2

Phylogenetic+Spatial: local+L1 speakers (nonlinear)		phylogenetic SD	0.02	0.17	0.73	288.39	L2
		spatial SD	0.34	0.57	0.85	288.39	L2
		Intercept	-0.02	0.11	0.25	288.39	L2
		social SD: L1	0.00	0.01	0.03	288.39	L2
Phylogenetic+Spatial: local+L1 speakers (linear)		phylogenetic SD	0.02	0.16	0.74	288.92	L2
		spatial SD	0.33	0.56	0.84	288.92	L2
		Intercept	0.01	0.13	0.25	288.92	L2
		L1	-0.17	0.05	0.28	288.92	L2
Phylogenetic+Spatial: local+L1 speakers (nonlinear)+L2 proportion (nonlinear)		phylogenetic SD	0.02	0.17	0.73	288.99	L2
		spatial SD	0.34	0.58	0.85	288.99	L2
		Intercept	-0.15	0.07	0.30	288.99	L2
		social SD: L1	0.00	0.01	0.03	288.99	L2
		social SD: L2 proportion	0.00	0.01	0.03	288.99	L2
Phylogenetic+Spatial: local+L1_log10:L2 proportion (linear)		phylogenetic SD	0.02	0.17	0.73	289.70	L2
		spatial SD	0.34	0.57	0.84	289.70	L2
		Intercept	-0.08	0.11	0.30	289.70	L2
		L1*L2 proportion	-0.13	0.02	0.17	289.70	L2

Phylogenetic+Spatial: local+L2 proportion (nonlinear)		phylogenetic SD	0.02	0.17	0.73	290.59	L2
		spatial SD	0.33	0.55	0.82	290.59	L2
		Intercept	-0.14	0.08	0.31	290.59	L2
		social SD: L2 proportion	0.00	0.01	0.03	290.59	L2
Phylogenetic+Spatial: local+L2 proportion (linear)		phylogenetic SD	0.02	0.17	0.72	290.76	L2
		spatial SD	0.32	0.55	0.82	290.76	L2
		Intercept	-0.03	0.16	0.36	290.76	L2
		L2 proportion	-0.98	-0.19	0.59	290.76	L2
L1 speakers (nonlinear)		Intercept	-0.05	0.12	0.29	309.88	L2
		social SD: L1	0.00	0.01	0.03	309.88	L2
L1 speakers (linear)		Intercept	-0.03	0.13	0.28	310.09	L2
		L1	-0.16	0.03	0.22	310.09	L2
L2 proportion (nonlinear)		Intercept	-0.13	0.10	0.33	310.48	L2
		social SD: L2 proportion	0.00	0.01	0.03	310.48	L2
L1_log10:L2 proportion (linear)		Intercept	-0.10	0.10	0.30	310.49	L2
		L1*L2 proportion	-0.11	0.03	0.16	310.49	L2
L2 proportion (linear)		Intercept	-0.05	0.15	0.36	310.89	L2
		L2 proportion	-0.83	-0.13	0.58	310.89	L2

L1 speakers (nonlinear)+L2 proportion (nonlinear)		Intercept	-0.14	0.09	0.33	312.53	L2
		social SD: L1	0.00	0.01	0.03	312.53	L2
		social SD: L2 proportion	0.00	0.01	0.03	312.53	L2
L1 speakers (linear)+L2 proportion (linear)		Intercept	-0.05	0.15	0.36	312.71	L2
		L1	-0.16	0.03	0.22	312.71	L2
		L2 proportion	-0.83	-0.12	0.59	312.71	L2

Table S3. Percentage of variance explained by phylogenetic and spatial random effects in top-ranking models of fusion and informativity incorporating random effects and the effects of the number of L1 speakers and Vehicularity.

response	sociodemographic predictor(s)	variance for the Gaussian observations in %	variance for phy_id in %	variance for sp_id in %	WAIC
fusion	L1 speakers	4	92	4	1,796.34
	L1 speakers + Vehicularity	4	92	3	1,798.71
	L1 speakers*Vehicularity	4	92	4	1,814.65
	Vehicularity	5	92	4	1,814.82
informativity	L1 speakers + Vehicularity	23	68	9	3,128.91
	L1 speakers	22	70	9	3,132.76
	L1 speakers*Vehicularity	25	66	10	3,135.58
	Vehicularity	23	68	9	3,136.39

Table S4. The quantiles (0.025, 0.5, and 0.975) of estimates of the fixed effect of the number of L1 speakers (log-transformed to the base of 10 and standardized to have a mean of 0 and variance of 1) in three models predicting morphological complexity scores (used in Lupyan & Dale (*6*)) without and with random effects. The differences in sample size depend on 1) whether the WALS languages with calculated morphological complexity scores are also available on the global tree and in Glottolog with specified location data (the prerequisites for including spatiophylogenetic effects in the model) and 2) whether the model was fit for all available languages or only those for which at least 35% of the relevant features are available in WALS. The default feature coverage threshold refers to the cut-off used in the Lupyan & Dale (*6*) study: at least 3 available features out of 28 (~10%) WALS features included in the morphological complexity metric.

model	2.5%	50%	97.5%	sample size	feature coverage threshold
morphological complexity ~ L1	-0.03	-0.01	0.01	448	default (~10%)

morphological complexity ~ L1 + phylogenetic effect + spatial effect	-0.05	-0.02	0	441	default (~10%)
morphological complexity ~ L1 + phylogenetic effect + spatial effect	-0.07	-0.03	0.02	77	35%

Table S5. WAIC values of spatiophylogenetic models of fusion and informativity with fixed and random effects using different priors

model	WAIC	prior	response
Phylogenetic+Spatial: local+L1 speakers (linear)	1,796.34	0.1	fusion
Phylogenetic+Spatial: local+L1 speakers (nonlinear)	1,797.20		
Phylogenetic+Spatial: local+L1 speakers (linear)+Vehicularity	1,798.71		
Phylogenetic+Spatial: local+L1_log10:Vehicularity	1,814.65		
Phylogenetic+Spatial: local+Vehicularity	1,814.82		
Phylogenetic+Spatial: local+Neighbours	1,818.78		
Phylogenetic+Spatial: local+Official	1,820.45		
Phylogenetic+Spatial: local+Education	1,821.36		
Phylogenetic+Spatial: local+L1 speakers (linear)	1,804.23	0.01	
Phylogenetic+Spatial: local+L1 speakers (nonlinear)	1,804.98		
Phylogenetic+Spatial: local+L1 speakers (linear)+Vehicularity	1,806.76		
Phylogenetic+Spatial: local+L1_log10:Vehicularity	1,822.47		
Phylogenetic+Spatial: local+Vehicularity	1,822.64		
Phylogenetic+Spatial: local+Neighbours	1,826.06		
Phylogenetic+Spatial: local+Official	1,827.50		
Phylogenetic+Spatial: local+Education	1,828.97		

Phylogenetic+Spatial: local+L1 speakers (nonlinear)	1,789.30	0.5	
Phylogenetic+Spatial: local+L1 speakers (linear)	1,790.71		
Phylogenetic+Spatial: local+L1 speakers (linear)+Vehicularity	1,792.96		
Phylogenetic+Spatial: local+L1_log10:Vehicularity	1,809.09		
Phylogenetic+Spatial: local+Vehicularity	1,809.27		
Phylogenetic+Spatial: local+Neighbours	1,813.10		
Phylogenetic+Spatial: local+Official	1,815.02		
Phylogenetic+Spatial: local+Education	1,815.94		
Phylogenetic+Spatial: local+L1 speakers (linear)	1,788.29	0.99	
Phylogenetic+Spatial: local+L1 speakers (nonlinear)	1,789.44		
Phylogenetic+Spatial: local+L1 speakers (linear)+Vehicularity	1,790.50		
Phylogenetic+Spatial: local+L1_log10:Vehicularity	1,806.72		
Phylogenetic+Spatial: local+Vehicularity	1,806.90		
Phylogenetic+Spatial: local+Neighbours	1,810.73		
Phylogenetic+Spatial: local+Official	1,812.70		
Phylogenetic+Spatial: local+Education	1,813.63		
Phylogenetic+Spatial: local+L1 speakers (linear)+Vehicularity	3,128.91	0.1	informativity

Phylogenetic+Spatial: local+L1 speakers (linear)	3,132.76		
Phylogenetic+Spatial: local+L1 speakers (nonlinear)	3,135.03		
Phylogenetic+Spatial: local+L1_log10:Vehicularity	3,135.58		
Phylogenetic+Spatial: local+Vehicularity	3,136.39		
Phylogenetic+Spatial: local+Neighbours	3,153.06		
Phylogenetic+Spatial: local+Education	3,156.96		
Phylogenetic+Spatial: local+Official	3,157.39		
Phylogenetic+Spatial: local+L1 speakers (linear)+Vehicularity	3,137.77	0.01	
Phylogenetic+Spatial: local+L1 speakers (linear)	3,140.58		
Phylogenetic+Spatial: local+L1_log10:Vehicularity	3,143.16		
Phylogenetic+Spatial: local+L1 speakers (nonlinear)	3,143.42		
Phylogenetic+Spatial: local+Vehicularity	3,144.23		
Phylogenetic+Spatial: local+Neighbours	3,160.68		
Phylogenetic+Spatial: local+Education	3,164.60		
Phylogenetic+Spatial: local+Official	3,165.34		
Phylogenetic+Spatial: local+L1 speakers (linear)+Vehicularity	3,124.09	0.5	
Phylogenetic+Spatial: local+L1 speakers (linear)	3,127.38		

Phylogenetic+Spatial: local+L1_log10:Vehicularity	3,130.25		
Phylogenetic+Spatial: local+L1 speakers (nonlinear)	3,130.36		
Phylogenetic+Spatial: local+Vehicularity	3,131.01		
Phylogenetic+Spatial: local+Neighbours	3,146.25		
Phylogenetic+Spatial: local+Education	3,150.27		
Phylogenetic+Spatial: local+Official	3,150.94		
Phylogenetic+Spatial: local+L1 speakers (linear)+Vehicularity	3,120.98	0.99	
Phylogenetic+Spatial: local+L1 speakers (linear)	3,125.07		
Phylogenetic+Spatial: local+L1_log10:Vehicularity	3,127.34		
Phylogenetic+Spatial: local+Vehicularity	3,128.16		
Phylogenetic+Spatial: local+L1 speakers (nonlinear)	3,128.37		
Phylogenetic+Spatial: local+Neighbours	3,144.52		
Phylogenetic+Spatial: local+Education	3,147.45		
Phylogenetic+Spatial: local+Official	3,148.42		

S1 Spatial effects

We tested three versions of spatial parameters. Two were based on precision matrices that incorporated spatial parameters that assumed diffusion/borrowing of features 1) at shorter distances, such several hundreds of kilometers ("local" set of parameters) and 2) at larger distances, such as several thousands of kilometers ("regional" set of parameters) (see Figure S1). Additionally, we fit random effects of group membership in one of the AUTOTYP areas. When examining top-ranking models of fusion and informativity, we find that these invariably include the spatial effects fitting the assumptions of the "local" version. This indicates that complex features associated with fusion and informativity are likely to be shared by languages that are not located too far from each other.

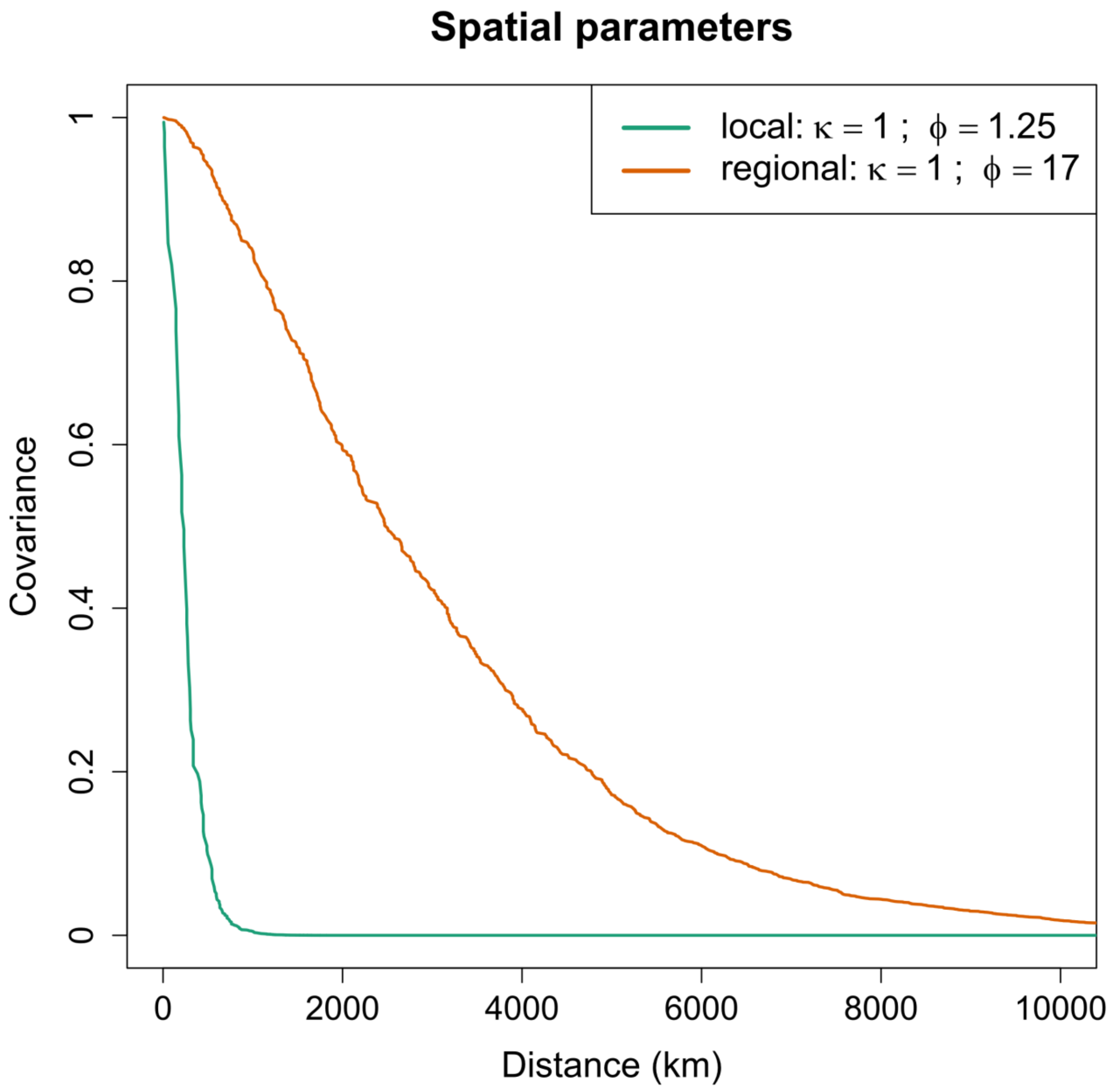

Fig. S1. The covariance in complexity scores decays with increasing distances between languages: covariance decays faster between languages on shorter distances under the local set of spatial parameters (green) than on the regional set (red). The local parameters κ (the additional smoothness parameter) and ϕ (the correlation function parameter) restrict the diffusion between

languages to distances below 1,000 kilometers, while under the regional parameters these distances can span several thousands of kilometers.
